# Proposal for a National Blueprint Framework to Monitor Progress on Water-Related Sustainable Development Goals in Europe

**DOI:** 10.1007/s00267-019-01231-1

**Published:** 2019-12-03

**Authors:** B. Essex, S. H. A. Koop, C. J. Van Leeuwen

**Affiliations:** 1 KWR Water Research Institute, Groningenhaven 7, 3433 PE Nieuwegein, the Netherlands; 2 Copernicus Institute of Sustainable Development, Utrecht University, Princetonlaan 8a, 3508 TC Utrecht, the Netherlands

**Keywords:** Sustainable development goals, Water management, Circular economy, Indicators, Implementation

## Abstract

The 17 Sustainable Development Goals (SDGs) underpinned by 169 targets presents national governments with huge challenges for implementation. We developed a proposal for a National Blueprint Framework (NBF) with 24 water-related indicators, centered on SDG 6 (clean water and sanitation for all), each with a specific target. We applied the NBF to 28 EU Member States (EU-28) and conclude that:

1. The current SDG 6 indicators are useful for monitoring progress toward water-related targets but their usefulness can be improved by focusing more on their practical implementation.
2. The extension of SDG 6 with complementary indicators (e.g. for the circular economy of water) and quantitative policy targets is urgently needed. This will benefit the communication process and progress at the science-policy interface.
3. SDG indicators can be improved in a *SMART* (specific, measurable, achievable, relevant, and time-bound) manner and by setting clear policy targets for each indicator, allowing for measuring distance-to-targets. This allows country-to-country comparison and learning, and accelerates the SDG implementation process.
4. We propose 24 water-related indicators centered on SDG 6, with complementary indicators including quantitative policy targets. The approach is doable, easily scalable, and flexibly deployable by collecting information for the EU-28.
5. Main gaps in the EU-28 are observed for water quality, wastewater treatment, nutrient, and energy recovery, as well as climate adaptation to extreme weather events (heat, droughts, and floods).
6. The framework was less successful for non-OECD countries due to lack of data and EU-centric targets for each indicator. This needs further research.

## Introduction

### Water Challenges and Sustainable Development

Water is crucial for human survival. It has been estimated that a minimum of 7.5 liters of water per person per day is required in the home for drinking, preparing food, and personal hygiene, the most basic requirements for water; at least 50 liters per person per day is needed to ensure all personal hygiene, food hygiene, domestic cleaning, and laundry needs (Hunter et al. 2010; Howard Bartram 2003). This however, does not include the amount of water used in agriculture, industry and which is required to maintain the Earth’s ecosystems. The global population is increasing from the current 7.7 billion to reach 8.5 billion by 2050, with over half of the population concentrated in less economically developed nations (United Nations 2015a). As these nations develop, the standard of living will increase and so with it the consumption of more water. Drinking water consumption in cities varies a lot. For instance, in Amsterdam and Copenhagen, the consumption is about 138 liters/person/day, whereas it is 622 liters/person/day in Bologna (Gawlik et al. 2017). Furthermore, in some areas overexploitation of groundwater may result in land subsidence and further increase the risks of flooding (Koop and Van Leeuwen 2017; Rahmasary et al. 2019).

Public water use is not the major freshwater use. In Europe, 44% of extracted water is used for agriculture and 40% for industry and energy production (EEA 2018). Detailed information about water withdrawal per sector is provided by the FAO (2016). Indirect water use via food consumption exceeds 3000 liters daily for most European Union (EU) citizens (Gawlik et al. 2017), meaning that actual agricultural water consumption exceeds 90% of total consumption (Hoekstra et al. 2012; Hoekstra 2014). With the increase in global population, the demand for food is expected to increase, added to this, the global trend toward a more meat-based diet will result in higher water consumption (Gawlik et al. 2017; Hoekstra 2014) and energy requirements per joule of energy in the food. To meet the increased food demand, land use is currently changing, from natural grassland and forests to agricultural land (Lambin and Meyfroidt 2011). The intensification of agricultural practices may increase with the use of agrochemicals to provide higher food yields. Loss of natural land reduces the area of natural water filtration and increases surface run off, increasing flood risk particularly in densely populated deltas and along the rivers and coasts. Use of chemicals in agriculture is a key contributor to water pollution (FAO 2018), the extensive amount of irrigation, and monocultures also lead to land degradation. Agriculture has direct environmental impacts on water quality, as well as indirect effects due to the increase in energy requirements. Both groundwater pumping for irrigation and increased agrochemical use result in increased water use and energy use per hectare (Rasul 2016), more industrial waste, as well as groundwater depletion (De Graaf et al. 2019).

Water is an important resource, with increasing demand but is also a critical requirement to the development of multiple sectors discussed above. With the end of the Millennium Development Goals (MDGs) in 2015 (United Nations 2015b), the Sustainable Development Goals (SDGs) were developed to continue the international agreement to sustainable development, this is known as the 2030 Agenda for Sustainable Development (United Nations 2015c). As water is so relevant, it was included as an individual Sustainable Development Goal (SDG) for Agenda 2030. Implementation matters (United Nations 2018).

### A Critical Reflection on the SDGs

#### History

The frequently cited beginning of the current sustainable development [rhetoric] is “Our common future” (Brundtland 1987). This narrative required economic development to occur as part of sustainable development (De Vries 2012; Robinson 2004). The Brundtland report also stressed the necessity of international cooperation for sustainable development. This conclusion increased awareness of the importance of sustainable development and led to further research and discussion. This gave rise to the MDGs for 2000–2015. The MDGs are focused on the development of the global south and have an anthropogenic focus of development. Whilst the MDGs had some large achievements (United Nations 2015b) the economic achievements were greater than the environmental goals (Georgeson and Maslin 2018). With the end of the MDGs in 2015, the SDGs were developed to continue the international agreement to sustainable development, this is known as the 2030 Agenda for Sustainable Development. Here the perception of sustainability has developed to include the requirements of environmental sustainability to achieving social and economic sustainability. The approach of the SDGs is *people, planet and prosperity* to include economic growth, environmental sustainability and social inclusion (United Nations 2015c).

**Fig. 1:**
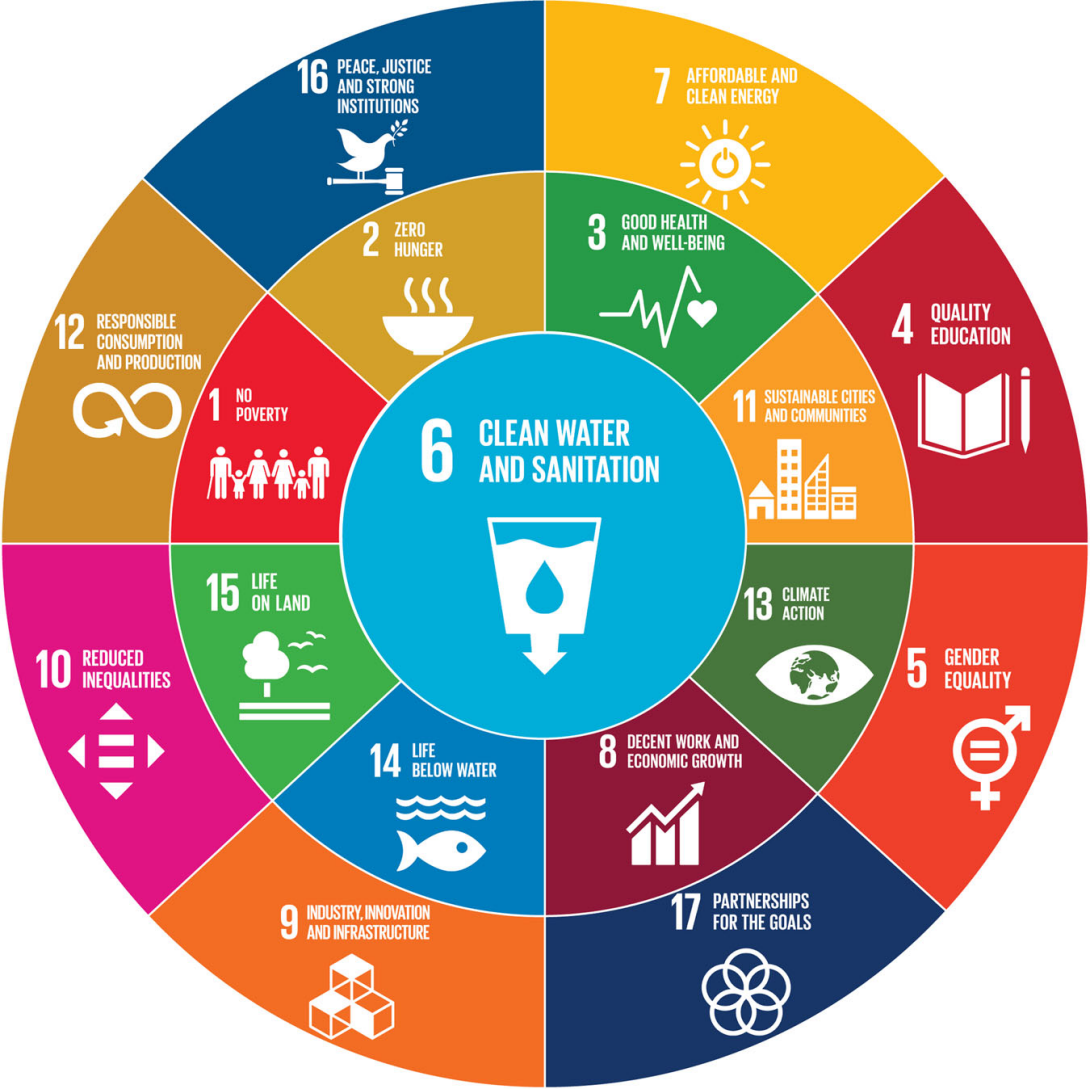
The water-centric 17 Sustainable Development Goals for each sector (United Nations 2015c; Makarigakis and Jimenez-Cisneros 2019; with permission)

Adopted in 2015, the SDGs form an internationally recognized set of goals and targets which aim to promote development in the economy, environment and society. There are a total of 17 goals (Fig. 1) containing 169 targets with a focus on *people, planet, prosperity, peace, and partnerships*. The SDGs aim for a larger, holistic approach than the MDGs. Although the SDGs have a higher number of targets, the number of indicators per target is less, with only 1.4 indicator per target compared with the 2.67 indicators per targets seen in the MDGs (Georgeson and Maslin 2018). In some respects, with so many targets, more indicators per target would require a large amount of monitoring.

A key learning point from the MDGs was that what gets measured, gets managed (Barnett 2015). The design of the indicators and what they measure affects what becomes developed and people aim to achieve higher results from the indicator monitoring (Bhaduri et al. 2016; Georgeson and Maslin 2018; Reidhead et al. 2016). Therefore, the lack of indicators may mean that the end goal targets are not met, and consequently more target-specific indicators may be beneficial. The indicators show progress to achieving the targets and thus the final goals, however in some cases the indicators are not sufficient to discern whether the target has been met (Weststrate et al. 2019). Target 6.1 for SDG 6 (water) is “universal and equitable access to safe and affordable drinking water for all” (Table 1). Therefore to be able to measure if this has been achieved, the data should be disaggregated and collected by age, gender, and income (Guppy et al. 2019). Many of the targets are nonnumerical and therefore even though indicator data is collected the end goal remains vague (Dickens et al. 2019) and the data can only be used as a comparative to other countries.

**Table 1:** The targets and associated indicators for SDG 6 of the SDGs (United Nations 2015c)

Targets	Indicators
6.1 By 2030, achieve universal and equitable access to safe and affordable drinking water for all	6.1.1 Proportion of population using safely managed drinking water services
6.2 By 2030, achieve access to adequate and equitable sanitation and hygiene for all and end open defecation, paying special attention to the needs of women and girls and those in vulnerable situations	6.2.1 Proportion of population using safely managed sanitation services, including a hand-washing facility with soap and water
6.3 By 2030, improve water quality by reducing pollution, eliminating dumping and minimizing release of hazardous chemicals and materials, halving the proportion of untreated wastewater and substantially increasing recycling and safe reuse globally	6.3.1 Proportion of wastewater safely treated 6.3.2 Proportion of bodies of water with good ambient water quality
6.4 By 2030, substantially increase water-use efficiency across all sectors and ensure sustainable withdrawals and supply of freshwater to address water scarcity and substantially reduce the number of people suffering from water scarcity	6.4.1 Change in water-use efficiency over time 6.4.2 Level of water stress: freshwater withdrawal as a proportion of available freshwater resources
6.5 By 2030, implement integrated water resources management at all levels, including through transboundary cooperation as appropriate	6.5.1 Degree of integrated water resources management implementation (0–100) 6.5.2 Proportion of transboundary basin area with an operational arrangement for water cooperation
6.6 By 2020, protect and restore water-related ecosystems, including mountains, forests, wetlands, rivers, aquifers, and lakes	6.6.1 Change in the extent of water-related ecosystems over time
6.A By 2030, expand international cooperation and capacity-building support to developing countries in water- and sanitation-related activities and programs, including water harvesting, desalination, water efficiency, wastewater treatment, recycling, and reuse technologies	6.A.1 Amount of water- and sanitation-related official development assistance that is part of a government-coordinated spending plan
6.B Support and strengthen the participation of local communities in improving water and sanitation management	6.B.1 Proportion of local administrative units with established and operational policies and procedures for participation of local communities in water and sanitation management

#### Trade-offs and synergies

Taking the indicators as individual measurements to work toward may have unexpected consequences. If a linear management approach is taken, progression toward one indicator, target or goal of the SDGs may result in a cancellation effect whereby progression toward another goal is then limited. Alternatively the development toward one indicator could result in a situation where one indicator then depends on the progress of another in order for development to occur (Scherer et al. 2018). These interlinkages occur due to feedback loops between the goals (Allen et al. 2018) and in some cases due to indicators being used for more than one goal (Pradhan et al. 2017).

The feedback loops between the goals lead to synergies, where development in one goal is beneficial to another (UN-Water 2016), as well as trade-offs (where development in one goal negatively impacts another). The goals that contain the most synergies are the social development goals: Poverty, zero hunger, good health, education, and gender equality (SDGs 1–5). Those with the highest number of trade-offs are economic growth and the environment (SDGs 8, 9, 12, 15; Pradhan et al. 2017). These interactions occur due to the current reliance of economic growth on increasing levels of consumption at the detriment to the environment. Work needs to be done in these areas to allow economic growth to detach from consumption. Without this, meeting the global population’s global needs and therefore meeting goals 1–3 will have a detrimental effect on the global use of water and land as well as increase the carbon emissions (Scherer et al. 2018) and lack of achievement of SDG 13, 15, and 6. The main problem of meeting people’s basic needs is that the level of consumption increases, however, progression toward a goal of responsible production and consumption (SDG 12) has the greatest trade-offs with meeting SDG 6 (Pradhan et al. 2017). These trade-offs must also be taken into consideration when creating policy to achieve the SDGs.

The separation of policy makers in different managerial departments often leads to policy becoming linear, with targets and actions for each indicator (Allen et al. 2018; Nilsson et al. 2016). To avoid this, an integrated approach is required to ensure that those actions that have synergies with other targets are implemented (Allen et al. 2018). This can often be hindered by the lack of technical capacity of skillsets of the policy makers. For this reason techniques are required to identify the goal synergies before policy is made, and frequent monitoring must occur to ensure that cancellation does not occur (Nilsson et al. 2016). The difference in national environment impacts the degree to which the synergies occur, and the impact that different actions have. For example in Nordic countries, biofuel does not have a negative impact on food production (Nilsson et al. 2016) and therefore could be used as an alternative energy source.

### The Implementation of the SDGs

#### SDGs and support for national water policies

An important aspect to consider is whether the collection of indicator data is beneficial to the nation collecting it. Continuing with the example above, the “access for all to a water supply” can be compared with other countries, however, for the individual nation, it does not provide data on the underlying reason behind the amount of access. An example given is that Ghana lacks water access due to lack of supply, whereas Nepal lacks access to water due to the level of contamination (World Bank 2018). Understanding the background reason on a national level provides an indication for the required water management.

#### SDG monitoring programs

Both Eurostat and the United Nations Environment Program (UNEP) have current SDG monitoring strategies. However, they both differ from the proposed study. Eurostat focuses on indicator trends for measuring the amount of change toward achieving the SDGs. The use of trends requires historic and current data and whilst Eurostat has both long-term and short-term trend data, the lack of historic data is still limited for all indicators of SDG 6 (except for 6.1 and 6.2), preventing the calculation of an overall trend score. In addition to this, the use of trends means that the Eurostat monitoring cannot use new indicators for recently measured data. The display of the Eurostat monitoring focuses on the results of the targets for each goal. Alternatively to this, the monitoring carried out by UNEP is for seven areas of environmental interest with each area having a selection of appropriate targets taken from different goals. Whilst this does work to highlight the synergies between goals, none of the seven areas specifically focusses on the management of water resources.

The Organization for Economic Co-operation and Development (OECD) has a set of SDG monitoring indicators that focus on the progression toward achieving the goals (OECD 2017, 2019a). Where there is no global data available for an SDG indicator, the OECD has identified an alternative indicator, however, in total, the indicators chosen by the OECD only evaluate 57% of the SDG targets (OECD 2017). Some of the missing indicators include those for water quality and transboundary water management amongst others meaning that SDG 6 is lacking coverage in these areas. The indicators are given as progression toward goals, where the target is either that given within an SDG target or it is the performance of the top 10% of OECD countries. Using the second target would mean that those countries in the top 10% would see no reason for further progression, even in situations where improvement could still occur.

#### SDG 6 and integrated water resources management

SDG 6 aims to address the increasing global problem of water scarcity with the aim to: *Ensure availability and sustainable management of water and sanitation for all* (United Nations 2015c). To enable this to happen eight targets have been agreed with nine associated indicators (Table 1).

Target 6.5 of SDG 6 is to implement Integrated Water Resources Management (IWRM) at all levels. IWRM is a policy making philosophy that aims to have a comprehensive and holistic approach to water management. However, what made IWRM popular was also part of its downfall; the broad scope, ambiguity of the concept and the lack of data made it difficult to create a holistic IWRM assessment framework for a national scale (Medema et al. 2008).

In order to assess the effectiveness of the IWRM and to provide feedback for decision makers, indicator frameworks are used. Many indicator frameworks focus on an individual problem such as the Water Stress Index and the Water Poverty Index (WPI). These indicators lack a holistic overview of the problem and in the case of WPI are complicated for policy makers to utilize (Petit 2016).

To decrease the complexity of IWRM indicators, some have chosen to focus on a specific geographic area such as the INBO Performance Indicators for African Basin Organizations or CAP-Net, a United Nations Development Program indicator for basin scale management.

Indicators on a city level include the City Blueprint Framework (CBF) which uses multiple performance orientated indices which report on different aspects of IWRM. The geometric average for each of the 25 indicators of the CBF forms the Blue City Index (BCI), which is available for more than 70 municipalities and regions in 40 different countries (Koop and Van Leeuwen 2015a, b). However, there is a lack of national level indexes that aim to give a holistic report on water management strategies.

In recent years IWRM has begun to lose its influence as an attractive management framework. There have been suggestions within the literature to move away from a one-size fits-all framework (Giordano and Shah 2014). This can be seen in the UNs’ World Water Development Reports which in 2012 placed IWRM as a central focus of the report, in 2015 it was scarcely present (Petit 2016).

### Knowledge Gap and Research Questions

Whilst the 17 individual SDGs are beneficial in that they together emphasize the extent to which sustainable development is interdisciplinary, they also result in isolating the different components of sustainable development. The further division of each goal into separate targets results in national managements strategies aiming for distinct targets within the goals rather than a cohesive development strategy. The problem of individualizing the goals is that many have conflicting interests which result in synergies and trade-offs between the ability to meet all the SDGs (Pradhan et al. 2017). The problem of this approach, which fails to highlight conflicting interests, is exemplified in water management. Although, water resources have their own target, SDG 6 focusses primarily on drinking water and sanitation, without appreciating the links between e.g. water, energy, agriculture, and health (Fig. 1).

Although there are theoretical approaches to managing water, often these are very challenging to achieve in practice (Savenije and Van der Zaag 2008). The governance actions required to meet the SDGs have been left to the individual nations. In fact, national governments conduct “only” voluntary national reviews. For many nations, this requires governance mechanisms to be strengthened (HLPW 2018) so that decisive actions can be taken (HLPW 2018) to reduce the risk of cherry-picking of the easiest goals to achieve. This uncoordinated approach may lead to international disputes and unanticipated side-effects.

There are many city-level indicators for sustainable living and water management (Hoekstra et al. 2018; European Commission 2015; Koop and Van Leeuwen 2015a; Siemens 2012), but alignment is required between national targets and the local performance. Globally, many countries lack consistent reporting strategies or the incentive to share data (Malik et al. 2015). This leaves many nations and international agencies uncertain about their progress toward the SDGs.

Many water-related indicators, whilst useful, do not show the scope of variables affecting water management. For example, the water scarcity index shows overuse of water, however, even if a country has water available, it may not be useable due to its poor quality—this is not shown with the water scarcity indicator. Conversely, with a more general indicator, there is a risk that the source of the development problem is lost (World Bank 2018). Therefore, there is a requirement for a water management index, which shows clear indicator results without over-simplifying the information on a national level. There is therefore, a need for a coordinated approach to water-management that incorporates indicators of multiple sectors for holistic SDG monitoring.

Based on the observed discrepancy between concepts such as IWRM that consider water as the principal unit of integration on the one hand, and SDGs and their specific targets on the other hand, the following research questions have been formulated: (1) What indicators can be used to create a water management framework for effective monitoring of progress in the EU-28? (2) What national indicators currently exist for water management assessment on a national level? (3) To what extent do these indicators align with the current SDG 6 targets and indicators? (4) Can a more suitable set of indicators be developed taking into account constraints of time and data availability? (5) To what extent does the proposed framework represent regional variability within European countries and non-EU countries?

## Development of a Framework of Water-related Indicators at National Level

### Literature Review

In determining what IWRM indicators are currently used at a national level a literature review was performed. A further in-depth assessment of the individual indicators used for the IWRM index was then carried out. This was done by entering each new indicator into a spreadsheet. The spreadsheet will count the number of times an indicator gets entered. Where indicators are essentially the same but are described differently, they would be grouped together. For example “water-related risk” from the sustainable city index (Batten 2016) and “exposure to floods and drought” from the TWAP-rivers indicators (UNEP-DHI and UNEP 2016) would be grouped together.

The assessment for the degree of indicator alignment with the SDG targets was carried out in two stages. A literature review was carried out to identify weaknesses and areas for improvement in the selected targets and indicators for the SDGs. This was done using Scopus with the search terms “SDG” AND water AND “goal 6” AND indicators, “SDG” AND water AND “goal 6” AND indicators, “SDG” AND “goal six” OR “goal 6”, “SDG” AND water AND “goal 6” AND indicators AND review, “agenda 2030” AND water AND indicators for the years between 2015 and 2019. This yielded a cumulative total of 217 papers, of these those relevant for review were then selected. The time frame was chosen partly to limit the responses. Following this review, a correlation analysis was then carried out between IWRM indicators entered into the database devised to answer question one and SDG target and indicators. The Pearson correlation coefficient (*r*) was used to assess the relationship between the IWRM indicators and the SDG indicators as well as the IWRM indicators and the SDG targets. The results were used to identify which indicators could be used for inclusion in the National Blueprint Framework (NBF).

### Identification of Suitable Indicators

**Fig. 2:**
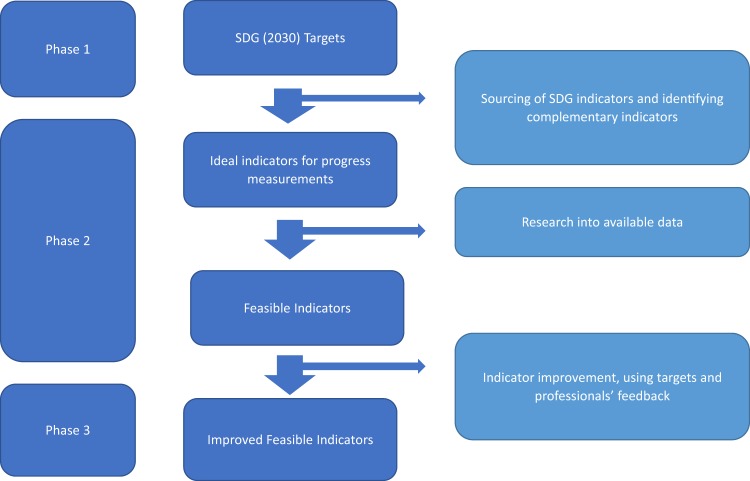
Flow diagram of the indicator development

After identifying the range of indicators used now, the indicators needed to monitor the water-related SDGs were identified and a set of more suitable indicators were then used to develop the NBF, following a similar approach to that used for the CBF (Koop and Van Leeuwen 2015a, b). The NBF developed is designed to provide a national level of water management monitoring toward the goals for Agenda 2030 and therefore the indicators used include those for the SDGs, but also complementary indicators. For each of the indicators specific targets were chosen, taken from those already present for the SDGs or a current EU target (European Commission 2017a). For those indicators for which no numerical target was available, a reasonable goal, based on scientific literature is proposed. The research process is shown in Fig. 2.

### Indicator Requirements

At present, some of the SDG 6 indicators have not been defined in a *SMART* manner. To ensure goals are clear and reachable, indicators should be *SMART*. Furthermore, indicators need to be simple so that they are useable and communicable to a range of practitioners, from industry, municipalities, governments, and Nongovernmental organizations (Table 2).

**Table 2:** Requirements for indicators (European Commission 2017b; Koop and Van Leeuwen 2015a)

Easy to access	Specific (simple, sensible, significant).
Easy to understand	Measurable (meaningful, motivating).
Timely and relevant	Achievable (agreed, attainable).
Reliable and consistent	Relevant (reasonable, realistic and resourced, results-based).
Credible, transparent, and accurate	Time bound (time-based, time limited, time/cost limited, timely, time-sensitive).
Developed with the end user in mind	

The indicators are chosen based on the measurement endpoints and practical limitations such as data availability. To determine whether water professionals would find the selected indicators useful, the proposed indicators were launched at the AIWW Summit in 2018. This resulted in some alternative suggested indicators, as well as the suggested progress-based approach. From this, the final set of indicators and their calculation methods was defined.

The main bottleneck in the development of indicators is to find reliable input (data) to calculate the indicator scores. In this study the focus has been on countries of the EU. This means that different specific sources of information can be used, e.g., data from Eurostat, data from the European Environmental Agency, and data from the OECD. For non-OECD countries, data are generally scarce and indicator development and calculations may be hindered or not be possible at all.

### Indicator calculation

The selected data for the indicators was available as both continuous values and pre-calculated numerical values. To be able to reach a final indicator value, two calculation steps had to be carried out. The first is to calculate the distance from the nation’s current raw data value to the target value. This gives a value for the progression toward the target. Following this, the progression value is then converted to a value between 0 and 10 to give the final indicator value. The value of 10 indicates that the target has been reached. Once the total number of indicators for a country had been collected, the geometric mean is calculated to find the National Blueprint Index (NBI) of the 24 indicators of the NBF. The geometric mean (Eq. 1) is used in preference to the arithmetic mean as it emphasizes the need to improve the lowest scoring indicators. The addition of plus one to each indicator score means that indicators with a zero value do not result in an index score of zero. This approach is similar to the City Blueprint Approach as developed by Koop and Van Leeuwen (2015a, b).

1 $${\mathrm{NBI}} = \root {{n}} \of {{\left( {{a}_1 + 1} \right) \times \left( {{a}_2 + 1} \right)...\left( {{a}_{n} + 1} \right)}} - 1$$

### Framework Analysis

Once the data had been collected the results were analyzed to determine if there were any dependencies between the indicators and whether the resultant scores made sense. The CBF was used to check the scores as it was assumed, given that the NBF was developed in a similar way, that there would be a linearity between the results. To be able to assess the dependencies between the indicators an internal indicator cross correlation was carried out. For this each indicator was measured against every other indicator and correlations compared. The assessment of the scores and the applicability of the indicator was done by selecting the identical CBF and NBF indicators for the countries that overlapped also using the Pearson’s rank correlation coefficient. In this analysis, both the correlation of country level results and the correlation between two similar indicators was carried out. The country level results were assessed to see if the end result showed a similar pattern to those seen at a city level. The correlation analysis used the Pearson calculation as it is applicable for this calculation because the dataset is linear and normally distributed.

## Results

### Indexes for Water Management

The review of IWRM frameworks revealed that the current IWRM indicators follow a diverse methodological approach. Some require data made available at a national level, others depend on gathering data via questionnaires. The indicators can measure the preventative steps in place, such as good governance and management frameworks, or investment in infrastructure like in the CBF (Koop and Van Leeuwen 2015a, b) and by the Asian Development Bank (2016). Indicators can also measure the current situation level, such as the age of sewer, or the problem such as the amount of water leakage.

Of the total IWRM frameworks identified in the literature review, nine were selected (Table 3) as the most relevant for further exploration into the indicators used. This yielded a database of 186 indicators that are currently used for IWRM. From the total of 186 indicators, only 13% of these were used more than five times. The most common indicators included access to water, access to sanitation, drinking water quality, and the level of secondary wastewater treatment (WWT). Whilst indicators assessed were predominantly performance related indicators, which measure the current state, the approach of the IWRM Indices depends on what they wish to achieve. For example, the Sustainable City Water Index has indicators for water balance and water reserves (Batten 2016) as this is monitoring how well a city manages water in a sustainable manner to not over consume resources. Conversely, the TWAP Index does not contain these indicators but has indicators such as political tension and legal frameworks within the river basin. This is due to the indicator measuring the impact of the water course being a transboundary resource and therefore includes the risk of potential resource conflicts. Both the TWAP Index and the Sustainable City Water Index contain an indicator for Water Stress as this relates both to sustainability and the potential for conflicts.

**Table 3:** The Indicator frameworks included in the indicator database

Indicator frameworks	Source
National Water Management Index	Asian Development Bank (2016)
City Blueprint Framework	Koop and Van Leeuwen (2015a, b)
Canadian Water Sustainability Index	Government of Canada (Policy Research Initiative 2007)
City Resilience Index	Arup (2014)
Environmental Performance Index	Wendling et al. (2018)
Global water security Index	Gain et al. (2016)
Sustainable City Water Index	Batten (2016)
SWESES	Kılkış (2018)
TWAP-rivers	UNEP-DHI and UNEP (2016)

When the IWRM indicators were compared with the indicators used for SDG 6, there was very little overlap with only 28% of the indicators in the IWRM indices also being used for SDG 6. The indicator for the degree of IWRM implementation (SDG 6.5.1) was not measured at all. However, the indices did contain indicators such as “management and action plans” or “effective management” which are less specific indicators of resource management. The lack of this indicator may be due to the indicator frameworks being used as part of an IWRM management plan, for example the Canadian Water Sustainability Index, and therefore it was not relevant to include IWRM implementation as an indicator.

Change in water-use efficiency, water stress, and the transboundary water cooperation agreement indicator (indicators 6.4.1, 6.4.2 and 6.5.2 respectively) were also lacking from many of the indices and each only occurred twice. The reason for the lack of inclusion may be due to multiple different facts such as lack of data availability, aggregation of data and a lack of perceived value. The efficiency of water use requires data to be gather over a number of years, which may have only recently begun. The transboundary management may have been included within the other management indicators and not disaggregated into a separate indicator.

### Alignment of the SDGs with Current IWRM Indicators

**Fig. 3:**
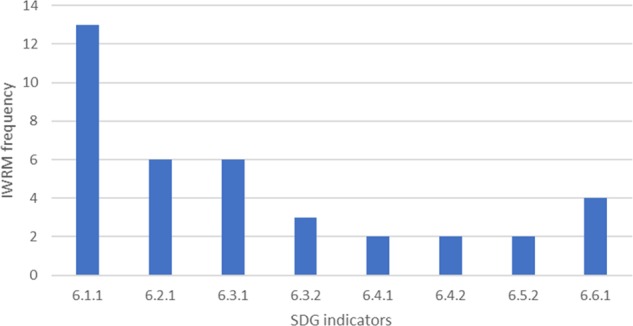
Number of IWRM indicators that align with the SDG indicators

Each IWRM Index is designed for a different setting, some having a river basin as the unit of analysis, some others a more urban or regional setting. IWRM can also be designed for disaster prone or less developed areas having indicators specific for that level of development. However, there are some indicators that are deemed to be important in multiple locations. These indicators also align with those required for SDG indicator monitoring. Of the 138 IWRM indicators assessed, only 38 were indicators that were also used in the SDGs. Of these, 78% included access to water within their index, however, the only disaggregation of this indicator is between the access to drinking water and the drinking water quality. The second most common indicators present in 67% of Indexes were access to adequate sanitation and proportion of wastewater safely treated. For most of the indexes, this included any wastewater treated with secondary or higher level of WWT. Only the CBF (Koop and Van Leeuwen 2015a) included further disaggregation. The presence of good ambient water quality was measured in the indexes by the groundwater quality. However, water quality, measured in the environment was an uncommon indicator and only occurred in 33% of indexes. The SDG indicators that were least represented were change in water efficiency over time and level of water stress. These were present in only 22% of the indexes. Figure 3 shows that there are some significant gaps in the SDG indicators that are also found in IWRM indexes. This highlights a lack of available data for the specific SDG indicators, rather than that they are deemed unimportant as, other indicators are used that still align with the SDG targets.

**Fig. 4:**
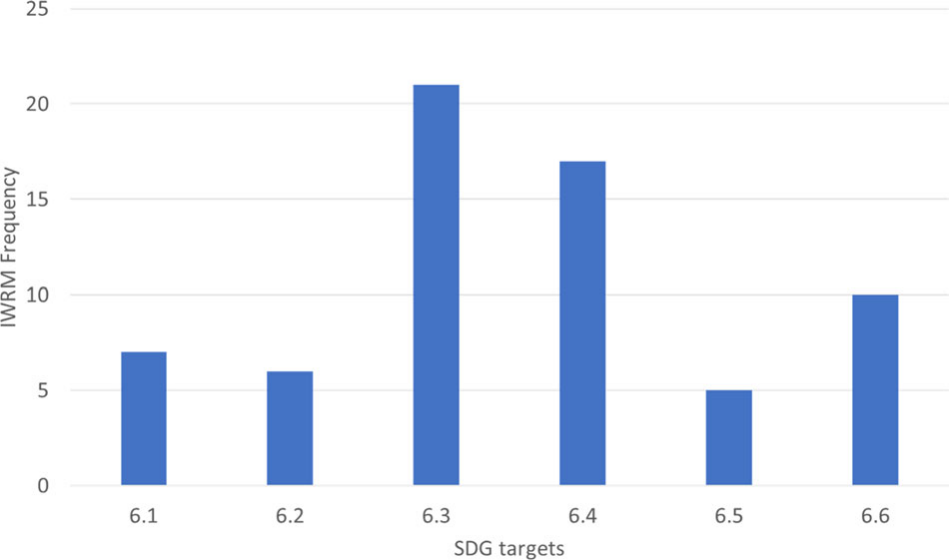
Number of IWRM indicators that align with the SDG targets

In total 66 indicators correlated with the SDG targets compared with only 38 indicators that correlated with the SDG indicators. A high number of indicators are relevant to SDG targets 6.3 and 6.4, 15% and 12.5%, respectively, but the specific SDG indicators are not commonly used. There is a smaller range of indicators that align with targets 6.1, 6.2, and 6.5 (Fig. 4). Of the total number of indicators that aligned with the SDG targets only 10% aligned with target 6.1, 9% with 6.2, and 8% for target 6.5. The indicators that aligned with 6.1 and 6.2 were identical for all indexes and were used by 78% and 68% of indexes respectively. This suggests that there is a high amount of data availability for the desired indicator and that there is cohesive thought in what indicator is needed. The lowest number of target and indicator synergies was seen with target 6.5. From the total number of indicators assessed, only 4% correlated with desired outcomes from target 6.5. The indicator only occurred in 44% of the total number of indexes assessed, and in one index, an indicator that aligned with target 5 occurred twice. This may be due to a lack of data or that water management is not perceived as an ideal IWRM monitoring measurement.

There is limited alignment between the SDG indicators and targets and the indicators produced for IWRM. This is in part because there are only 11 indicators for SDG 6, whereas all of the IWRM indicators assessed had greater than 15 indicators. The fact that only 28% of indicators align with the SDG indicators is in part due to their being more indicators per index initially. This is supported by the increased alignment of IWRM indicators with the SDG targets at 49% of indicators aligning. Where there is a lack of indicator alignment, an increase in target alignment is seen. This suggests that where there is a lack of available data the water management professionals find varying indicator alternatives for the same area of water management. This indicates that the themes seen as being required to monitor water management are cohesive between water professionals and those who designed the SDGs.

### Indicator Development, Selection, and Application

#### The selection of indicators

SDG 6 has only 11 indicators (Table 1), suggesting that there are many areas where complimentary water indicators would provide further information on the progression toward healthy water and sanitation. The IWRM indicators were used to provide a set of possible indicators for each target. From this an “ideal” set of indicators was selected according to the best options for measurement end goals. Data availability limited the use of some of these indicators and therefore a second set of feasible indicators was created for which data is available. The limitations in data availability were primarily in monitoring water efficiency and climate mitigation measures. A reliable data source was also necessary, the most readily available indicators were those collected by the UN due to its global coverage and reliability. There was a lack of data for water quality and water infrastructure management. Other indicators that were lacking or difficult to source include those for progression toward a circular economy. In some cases, calculation from the available data can provide a proxy indicator for this measurement. Once completed, this set of feasible indicators was sent for review by water professionals to check their opinion on the indicators relevance and meaningfulness. The indicators suggested by the review team (see “Acknowledgements”) included ecological water quality being separated from the surface water quality indicator as well as the inclusion of an indicator for water affordability. The final set of NBF indicators, divided into seven categories, is shown in Table 3.

To be able to show progression of each of the indicator it is required to have a specific target. Many targets and goals developed for agenda 2030 are not numerical. Targets 6.1 and 6.2 include the phase “access for all”, which has been assumed to mean 100% coverage. Target 6.3 aims to “improve water quality” without a numerical target given and target 6.4 aims to “substantially increase water-use efficiency”. Whilst this means that countries with different levels of development can all aim to achieve the targets, it does not provide a goal to aim for. For indicators with no clear goal, the next step was to check European targets. Only 16% or the indicators have useable SDG targets, compared with 38% of the indicators for which there was an EU target. The remaining indicators have targets supported by the literature. Table 4 summarizes the targets chosen for each NBF indicator.

**Table 4:** Final set of NBF indicators, targets and their reasoning

Category	Indicator	Target	Reasoning
I. Water stress	1. Water scarcity	20% of renewable water sources	The 20% of renewable source is the indicator set by the EEA of what is a sustainable amount of extraction. Achieving this indicator ensures sustainable rates of water extraction.
	2. Flood Vulnerability	Low risk	With the increased risk of flooding for Europe, the target for achieving low flood risk would indicate sufficient flood prevention investment.
	3. Transboundary cooperation	Low risk	Ensuring that all rivers gain a score of 1 regarding the Legal agreements for transboundary rivers according to the TWAP research assessment.
	4. Tertiary education attainment	40% of 25–64 years old	The EU target is 40% of 30–34 years old individuals have tertiary level education, due to data availability, the age spectrum has been widened. The authors consider tertiary education essential for good water governance (Romano and Akhmouch 2019; Koop et al. 2017)
II. Water quality	5. Surface water quality	Good score	The target is progression toward achieving a good score according to the WFD.
	6. Groundwater quality	Good score	The target is progression toward achieving a good score according to the WFD.
	7. Ecological water quality	Good score	The target is progression toward achieving a good score according to the WFD.
III. Access to basic services	8. Drinking water quality	100%	This indicates that the quality of water supplied is good for human consumption without further filtering.
	9. Drinking water connection	100%	Indicates the population connected to a drinking water supply within their home.
	10. Sanitation connection	100%	This indicates the percentage of the population connected to safely managed, nonshared sanitation facilities. The highest level of sanitation measurement by the JMP for the SDGs (JMP 2017).
	11. Water affordability	100% affordable <4% income	Whilst increasing water tariffs is a way of reducing water consumption, this indicator monitors whether the water remains affordable for the population.
IV. Infrastructure	12. Infrastructure investment	3.8% GDP	In order to ensure current infrastructure is maintained and developed, the level of infrastructure investment needs to be 3.8% GDP according to McKinsey and company.
	13. Water leakage (%)	0%	This indicates the quality of water infrastructure. 0% leakage would indicate efficient water usage.
V. Wastewater treatment	14. Secondary WWT (%)	100%	This indicates the number of countries that have reached secondary wastewater treatment (WWT).
	15. Tertiary WWT (%)	100%	This indicates those countries which are improving their water usage to allow reuse and fit with the target of achieving a more circular economy.
	16. Nutrient recovery (%)	100%	This indicates the amount of nutrients reclaimed from the used water. For a circular economy to be achieved all nutrients must be recovered.
	17. Waste water to energy	100%	This indicator shows the development of energy capture from wastewater. This can show developments in the standard of wastewater processing, and energy efficiency of the water cycle.
VI. Solid waste (SW) treatment	18. SW generated	10% less than 2010 levels	The target for Europe is to prevent waste being produced, as well as to increase recycling. The target of 10% less waste produced than 2010 quantities is the Spanish target for 2020 and has been set as the European standard.
	19. SW recycled (%)	65% total	This indicator shows the progression toward the EU 2030 recycling target.
	20. SW to energy (%)	100%	This indicator shows the progression toward achieving a circular economy by gaining energy from waste.
VII. Climate adaptation	21. CO_2_ emission per capita	32% of 1990 levels	The current European targets to reduce the CO_2_ emission to 27% lower than 1990 levels.
	22. Renewable energy (% of total)	32%	The European policy to reduce waste by 2030 includes a recycling target of 40% of total waste by 2030. This indicator can also show progress toward material use for the circular economy.
	23. Notre Dame Readiness Index	100%	The Notre Dame Readiness Index scores progression toward readiness to climate change. The highest level of “readiness” is 100.
	24. IWRM	100%	The percentage of IWRM. This shows progression toward SDG target 6.5.1.

#### Linking the selected indicators with the SDGs

Each of the indicators aligns with a specific target, some of the indicators are developed from the SDG indicators for which there is data available. The remaining indicators are complementary. The alignment of the indicators with the SDG targets can be seen in Table 5.

**Table 5:** SDG 6 and links between the key NBF indicators and complementary indicators and goal targets

NBF indicators	SDG direct goal link	Indirect SDG 6 link	SDG goal interlinkages
1. Water scarcity	6.4.2		16, 8, 2
2. Flood vulnerability			15, 13
3. Transboundary cooperation		6.5.2	17
4. Tertiary education attainment			4, 9
5. Surface water quality	6.3.2		15, 3
6. Groundwater quality	6.3.2		15
7. Ecological water quality	6.3.2		3, 15
8. Drinking water quality	6.1.1		3, 2
9. Drinking water connection		6.1	3, 15, 5, 4
10. Sanitation connection			3, 5
11. Water affordability			1, 10
12. Infrastructure investment		6.2	9
13. Water leakage (%)		6.4	9, 11, 12
14. Secondary WWT (%)	6.3.1		3, 11
15. Tertiary WWT (%)		6.3	3, 11
16. Nutrient recovery (%)			11, 12
17. Waste water to energy			7, 11
18. SW generated			11
19. SW recycled (%)	12.5		11, 13, 12
20. SW to energy (%)			7, 11, 12
21. CO2 emission per capita			13
22. Renewable energy % total			7, 11, 13
23. Notre Dame Readiness Index			13
24. IWRM	6.5.1		17, 12

#### Results for the EU-28

**Fig. 5:**
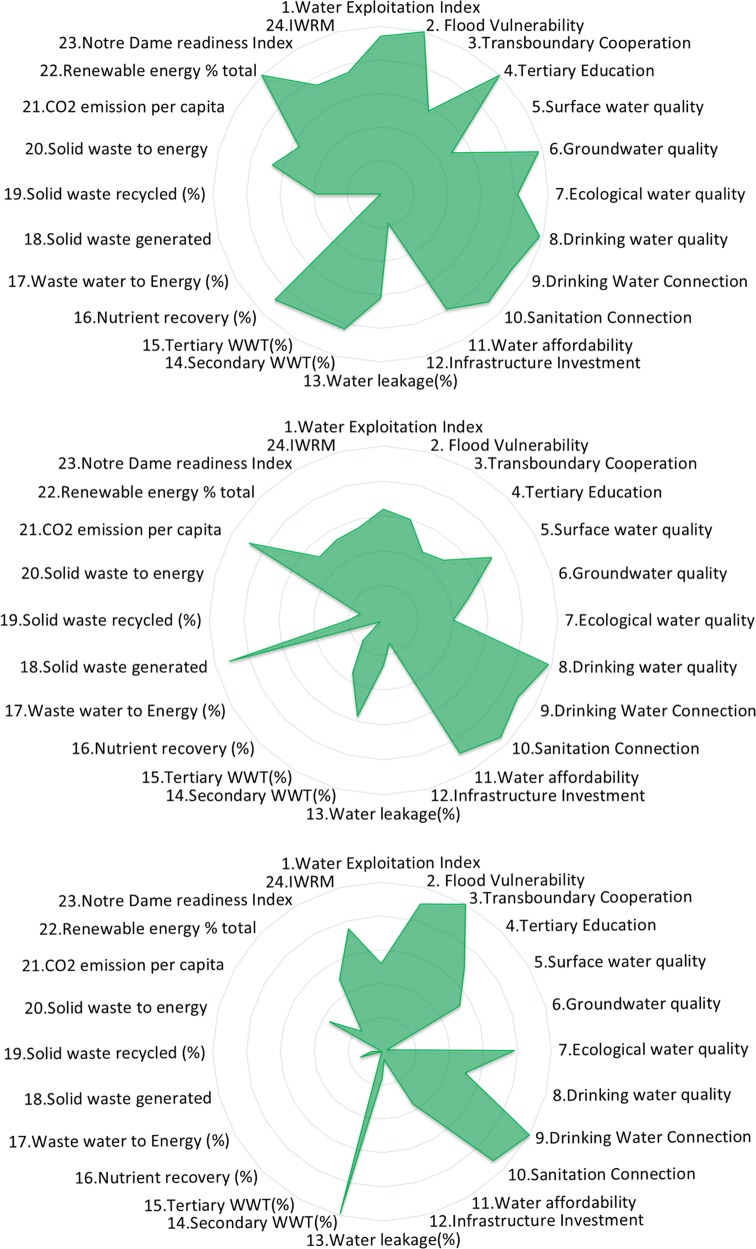
Indicator scores for the NBF for Finland (top), Italy (middle), and Malta (bottom) they receive an NBI score of 5.8, 4.5, and 2.6, respectively

**Fig. 6:**
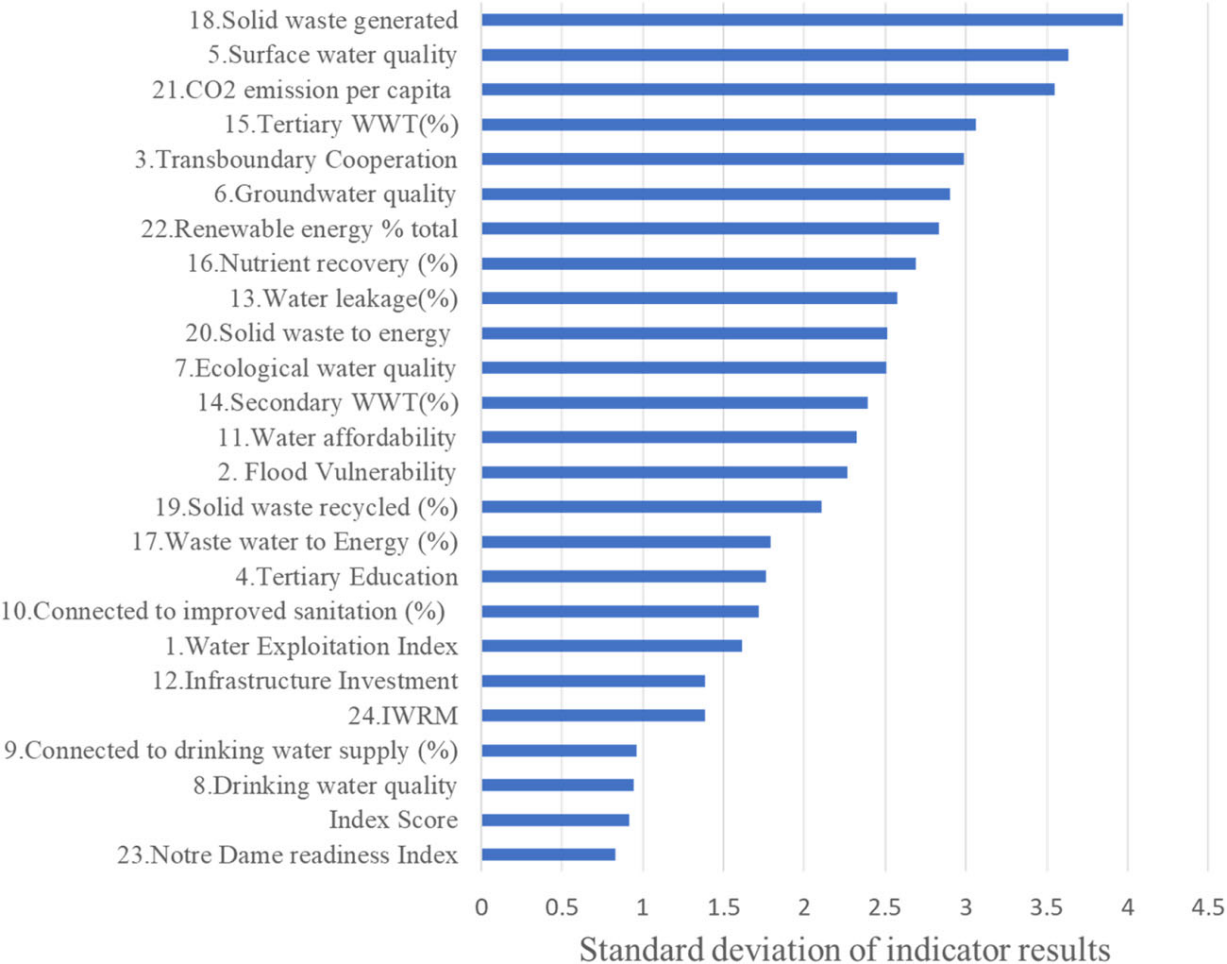
Standard deviation of indicator results among the EU-28

**Fig. 7:**
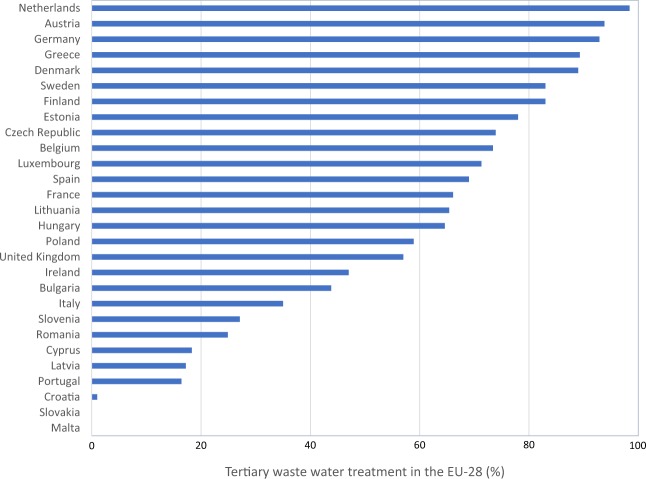
Comparison of tertiary wastewater treatment (%) among the EU-28

Details of the calculation of all indicators and the NBF spider diagrams for the EU-28 are provided in Supplementary Material. The results for the indicator scores of three EU countries is shown in Fig. 5. For the framework to be useable, it needs to clearly show the differences between the EU countries assessed. To check this the standard deviation of the results was calculated and shown in Fig. 6. The indicator showing the highest variability among the EU-28 is SW generated. The score with the least variability is the Notre Dame Readiness Index. This may be due to the global scale of the Notre Dame Readiness Index compared with the SW generated. An example of an indicator with a high variation between the EU-28 is tertiary WWT (Fig. 7). This provides great opportunities for strengthening international collaboration on IWRM between the EU-28, i.e., country-to-country learning. Other indicators that show low variability include the connection to drinking water supply and drinking water quality. There is an overall high score for drinking water and sanitation connections, however, in some countries with generally lower scores, there is a trend for sanitation to score lower than drinking water connection. It can also be concluded that more progress is needed toward energy and nutrient recovery to meet the circular economy ambitions in Europe (Van Leeuwen et al. 2018; Van Puijenbroek et al. 2019). The lowest overall NBI score was for Malta, and the highest overall NBI was for Finland. Malta also has the largest deviation in the indicator results, whereas France has the most consistent scoring (see Supplementary Material).

## Discussion

### Development of the NBF

The NBF is a proposal for a set of progress-based indicators that can help the countries in achieving the SDGs. The indicators chosen stem from current IWRM indicators, including those for the SDGs. The set of proposed water-related indicators of the NBF reaches a total of 24 indicators that are grouped into seven categories as shown in Table 4. The aim of the NBF is to use SDG 6 targets and indicators and to provide complementary indicators relevant for the water sector. We collected information for the EU-28 to demonstrate their implementation.

The popularity of indicators for policy implementation has led to an extensive use of indicators for a wide range of contexts (see Table 3). The initial search showed that indicators frameworks had been previously reviewed, in terms of their applicability for sustainable cities (European Commission 2015; Hoekstra et al. 2018) as well as their for usability as a sustainability indicator (Pires et al. 2017). However, there was a lack of information of frameworks applied at a national level and neither of the reviews focused on the use of the indicator frameworks in achieving the SDGs. To develop an overview of national level IWRM frameworks, a separate review was therefore carried out.

Due to the volume of indicators available and the time constraints of the project only nine IWRM frameworks were fully reviewed. These indicators contained 186 indicators, but only 35% were relevant to the SDGs. The review of the indexes was not limited by the perspective or the goal of the index. For instance, the TWAP reviewed the water management with a focus on issues which would cause conflict to transboundary water sources, such as water stress and poor governance. Conversely many of the other indexes had a far more introspective focus on national or city-based issues such as community involvement and performance of service utilities. When the IWRM indicators were compared against the SDG indicators it became apparent that indicators that had been used within the MDGs, such as Access to improved drinking water (MDG 7) were more popular than those not included such as water efficiency (SDG 6.4.1) and water management (SDG 6.5.1). For those already collected as part of the MDGs, it is likely that there is already easily accessible data available for these indicators and hence the wide usage of specific indicators.

### Strength and Weaknesses of the SDGs

The SDGs are an important step toward sustainability, resilience, and social security. Many of the SDGs are water-related (Fig. 1), but they are currently difficult to implement, mainly because the targets and indicators (Table 1) have not been developed in a *SMART* manner (Table 2). Other shortcomings have been identified as well. For instance, Weststrate et al. (2019) noted that the indicator of “access to an improved water source” fails to take water quality into account. They also noted that the indicator of “access to an improved sanitation facility” does not take into account safe collection, treatment and disposal of wastewater and fecal sludge, vital to disease prevention. The absence of clear targets are hindering effective implementation. The inclusion of targets for the indicators allows the measurement of progress toward a goal rather than the current performance of the nation. Showing progress toward a goal increases the use of the indicator as policy makers can discern whether they are on track to meet a target. Whilst the SDGs have 169 targets, very few of these have precise numerical goals. For those targets that did have numerical goals, these were chosen as the end target. The Sustainable Development Scenarios discussed at the Rio+20 United Nations Conference on Sustainable Development (United Nations Department of Economic and Social Affairs 2013) equally do not have many specific numerical targets for water and those that do, such as the target to give another 230 million people access to an improved water source is a target on a global rather than national scale and therefore difficult to apply nationally as the target is not relevant to every nation. The conclusion is that next steps are needed to improve the SDGs. The selection of water-related indicators and targets as proposed in this manuscript should be considered only as an example. It shows that it is possible to improve the SDGs, to better implement them, and that this is doable. We fully realize that other scientists may have arrived at another set of indicators. Anyhow, it is not up to scientists to decide on which indicator and targets should be selected but to the science-policy arena.

### Data Availability

The proposed NBF was also applied to some non-EU countries (South Korea, USA, Tanzania and Brazil) to test its global relevance. The largest problem found in applying the NBF was the lack of data. In total, data for 16 indicators could be found for South Korea and the United States, with only 13 indicators having the required data for Tanzania, this number decreased to only 12 for Brazil. The larger number of indicators found for South Korea and Brazil was due to them being part of the OECD and therefore present in those datasets. In some cases, alternative proxy indicators were used, such as for water access to drinking water, the EU countries used “proportion of the population using improved water supplies” which were under the category of ‘safely managed’ from the World Health organization. However, for South Korea, Brazil, and Tanzania, there was no data available for ‘safely managed’ and instead, “piped” category was used instead. In other instances, the data was not available from the source used for the EU and was instead found from other data sources. To compare index scores of the non-EU countries, the indicators for which every country had data were separated. From the indicator scores, the geometric mean was calculated to create an index for every country. The Index scores were then ranked from 1 (being highest) to 32 (being lowest). South Korea ranked 28th but the United States, Brazil, and Tanzania ranked 30th, 31st, and 32nd, respectively.

The points above suggest that more data needs to be collected and made readily available for global datasets and what gets measured will get managed (Georgeson and Maslin 2018), it is important that to achieve the SDGs for water, good governance needs to occur and so also data collection for the indicators. However, excess collection may put undue pressure on the nation and take resources away from other areas of management (Dickens et al. 2019).

### Problem of Scale and Perspective

The SDGs are designed to be implemented on a global scale and as such, the indicators need to be applicable to every country. This is highlighted in indicators such as SDG 6.1.1 Proportion of population using safely managed drinking water services and 6.2.1 Proportion of population using safely managed sanitation services, including a hand-washing facility with soap and water. In the EU, these average at 91% and 80% achievement, respectively. Whilst there is still room for improvement, these indicators do not help to determine the cause for the lack of these services or the degree to which these services are efficient and sustainable. If it did, those countries scoring highly could identify where there is still room for improvement. This leads to the problem of not every country having the same starting point (Equal Measures 2030 2019) with the global south having more progress required in social indicators, whereas the global north has a greater focus on achieving the environmental indicators.

The framework was developed from the SDGs but also a set of indicators that primarily were sourced from initiatives in the global north, which leads to a bias within the framework of indicators relevant to the state of development within the EU. The impact of this on the developed framework can be seen when it is applied to the non-EU countries, where Tanzania scores 10 for renewable energy % (NBF indicator 22) but only 3.5 for Connected to drinking water supply (NBF indicator 9). Some of the indicators are related to the circular economy of water and measured in a way that works with European infrastructure, this assumes a European model of development. As well as the indicators being relevant to Europe, the targets too are based on the EU goals, and targets that could be realized in this situation. However, as not all countries have the same starting point, it would not be realistic to assume they could achieve the same end goal in 15 years.

The variation in the NBI for the EU-28 is small. The NBI varied from 2.6 (Malta) to 5.8 (Finland) as shown in Fig. 5. The BCI allows a comparison of the municipalities and regions per country (Gawlik et al. 2017). The differences in the BCI among the municipalities is relatively small. In this regard the NBF may be indicative for a country to monitor progress toward SDG 6, but often, solutions have to be created at the level of regions and municipalities, where most of us live (Koop and Van Leeuwen 2017). For instance, for ten municipalities assessed in the Netherlands the BCI varied from 5.7 to 8.3. For the UK, with five municipalities and regions assessed, the BCI varied from 5.3 to 6.7. For Sweden with five municipalities assessed the BCI ranged from 6.9 to 7.8. For the USA, with six cities assessed, the BCI ranged from 3.9 to 5.4 (Feingold et al. 2018).

Based on the research carried out, we propose an NBF with 24 water-related indicators centered on SDG 6, with complementary indicators (e.g., to monitor goals related to the circular economy of water) including quantitative policy targets. The NBF indicators are complementary to the CBF indicators to measure water management performance at municipal and/or regional level. This is where probably most of the efforts are needed.

We realize that in the end the selection of indicators and their quantitative targets are political decisions. We also realize that the collection of data can be a burden for many non-OECD countries, but informed decision-making and exploring synergies or co-benefits is the way forward (Koop and Van Leeuwen 2017). If “what gets measured gets managed, measuring the wrong thing” or neglecting to measure at all, really matters (Barnett 2015; Georgeson and Maslin 2018). In our view it is better (and certainly cheaper) to do an in-depth diagnosis on SDG 6, than to implement regrettable transitions (UNEP 2013) to modernize and expand the world’s urban water infrastructure as the projections of global financing needs for water infrastructure range from USD 6.7 trillion by 2030 to USD 22.6 trillion by 2050 (OECD 2019b). Looking at the crucial role of water in the SDG framework (Fig. 1), quoting former Prime Minister Wim Kok of the Netherlands is quite appropriate here as he once said: “a priority is only a priority if you give it priority”.

## Conclusions

1. The current SDG 6 indicators are useful for monitoring progress toward water-related targets but their usefulness can be improved significantly by focusing more on their practical implementation.
2. The extension of SDG 6 with complementary indicators (e.g. to monitor goals related to the circular economy of water) and quantitative policy targets is urgently needed. This will benefit the communication process of monitoring SDG 6 progress at the science-policy interface.
3. The implementation of the ambitious SDGs can be improved by reformulating the indicators in a *SMART* manner and by setting clear policy targets for each of the indicators, allowing for measuring distance-to-targets. In this way international country comparisons can be made.
4. We propose 24 water-related indicators centered on SDG 6, with complementary indicators including quantitative policy targets. We realize that in the end indicators and quantitative targets are political decisions.
5. We demonstrate that this approach is doable, easily scalable, and flexibly deployable by collecting information for the EU-28.
6. Main gaps at national level in the EU-28 are observed for water quality, WWT, nutrient, and energy recovery, as well as for climate adaptation to extreme weather events, e.g. extreme heat, droughts, and floods. This provides options for further international collaboration or country-to-country learning to speed up the SDG implementation process.
7. Whilst the NBF scores for the EU-28 correlate with municipal and regional water management performance, the framework was less successful for non-OECD countries due to the restricted data availability and EU-centric targets for each indicator. This needs further research.

## Supplementary information

Supplementary material

Indicator supplement

## Abbreviations

BCI: Blue City Index
CBF: City Blueprint Framework
EU: European Union
EU-28: 28 Member States of the European Union
IBNET: international benchmarking network for water and sanitation utilities
IWRM: integrated water resources management
JMP: WHO/UNICEF Joint Monitoring Program for Water Supply, Sanitation and Hygiene
NBF: National Blueprint Framework
NPI: National Blueprint Index
MDGs: Millennium Development Goals
OECD: Organization for Economic Co-operation and Development
SDG: UN Sustainable Development Goal
SDG 6: SDG to ensure availability and sustainable management of water and sanitation for all
SW: solid waste
SMART: acronym, giving criteria to guide in the setting of objectives. The letters S and M generally mean specific and measurable. The remaining letters refer to achievable (or attainable), relevant, and time-bound
TWAP: Transboundary Water Assessment Program
UN: United Nations
UNDP: United Nations Development Program
UNEP: United Nations Environment Program
WFD: Water Framework Directive
WHO: World Health Organization
WPI: World Poverty Index
WWT: wastewater treatment
