# Supplementary material for: Proposal for a National Blueprint Framework to Monitor Progress on Water-Related Sustainable Development Goals in Europe
Source: Environ Manage. 2019 Dec 3;65(1):1–18. doi: 10.1007/s00267-019-01231-1 (PMC6960216; doi:10.1007/s00267-019-01231-1)

SUPPLEMENTARY MATERIAL

PROPOSAL FOR A NATIONAL FRAMEWORK TO MONITOR PROGRESS ON WATER-RELATED SUSTAINABLE DEVELOPMENT GOALS IN EUROPE

ESSEX B, KOOP SHA AND VAN LEEUWEN CJ

The supplementary material consists of three separate annexes:

- Annex 1. Indicators of the National Blueprint Framework
- Annex 2. Results of some of the indicator scores for the 28 EU Member States
- Annex 3. Spider diagrams of the NBF for each of the 28 EU Member States

Annex 1. Indicators of the National Blueprint Framework (NBF)

First a general description is given, followed by a specific example of each of the 24 indicators of the NBF.

A. General description

To be able to reach a final indicator value, two calculation steps have to be carried out. The first is to calculate the distance from the nation's current raw data value to the target value. This gives a value for the progression towards the target. Following this, the progression value is then converted to a value between 0-10 to give the final indicator value. The value of 10 indicates that the target has been reached (Figure 1).

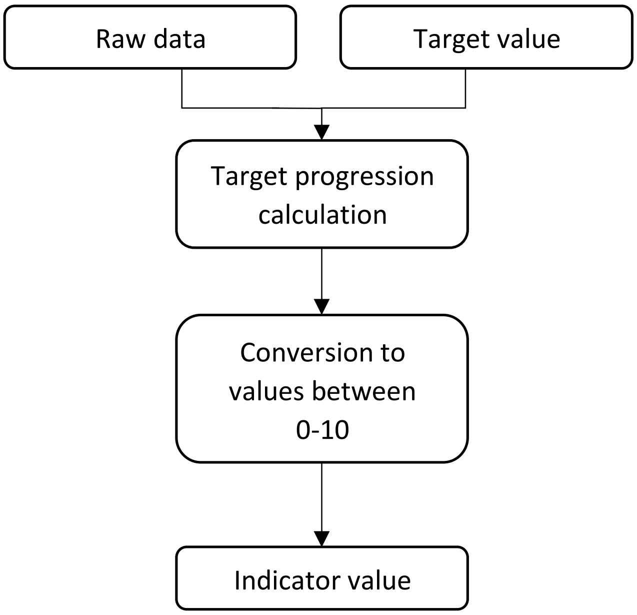

Figure 1. *Flow diagram to show the steps taken to reach the final NBF indicator score from the original data value.*

In these examples raw data values in percentages, with an end target of 100, where A donates the raw data value, progression is the percentage value, and to convert to a value between 0-10 it is divided by 10. In cases where the end target is 100% the calculation is shown below.

$$\mathbf{Indicator} = \frac{\mathbf{A}}{\mathbf{10}}$$

If instead the target is 0%, the following calculation is carried out.

$$\mathbf{Indicator} = \mathbf{1} - \frac{\mathbf{A}}{\mathbf{10}}$$

For continuous values, or those in percentages where the target is a value other than 100, the values are multiplied by a conversion factor to result in a range between 0-10, where scoring 10 means that the target has been reached. In the following example A gives the national raw data value and B gives the target value. C is the conversion factor.

$$\textbf{Indicator} = \frac{\boldsymbol{A}}{\boldsymbol{B}} * \boldsymbol{C}$$

If the indicator calculation included the progression towards a target value, a minimum-maximum calculation was used. With A being the minimum value and B being the target value and X being the country value.

$$\textbf{Indicator} = \frac{(\boldsymbol{X} - \boldsymbol{A})}{(\boldsymbol{B} - \boldsymbol{A})}$$

Once the total number of indicators for a country had been collected, the geometric mean could be calculated to find the final index value of the NBF. The geometric mean is used in preference to the arithmetic mean as it reduces the impact of high or low scores. The addition of plus one to each indicator score means that indicators with a zero value do not result in an index score of zero. The approach is similar to the method developed by Koop and van Leeuwen (2015, a, b).

$$\textbf{National Blueprint Index} = \sqrt[n]{(\boldsymbol{a_1} + \mathbf{1}) \times (\boldsymbol{a_2} + \mathbf{1}) \ldots (\boldsymbol{a_n} + \mathbf{1})} - \mathbf{1}$$

Table 1 – *Overview of the 24 indicators of the NBF*

Category	Indicator
**Water stress**	1. Water scarcity
	2. Flood Vulnerability
	3. Transboundary cooperation
	4. Tertiary education attainment
**Water quality**	5. Surface water quality
	6. Groundwater quality
	7. Ecological water quality
**Access to basic services**	8. Drinking water quality
	9. Drinking water connection
	10. Sanitation connection
	11. Water affordability
**Infrastructure**	12. Infrastructure Investment
	13. Water leakage (%)
**Waste water treatment**	14. Secondary WWT (%)
	15. Tertiary WWT (%)
	16. Nutrient recovery (%)
	17. Waste Water to Energy
**Solid waste treatment**	18. Solid waste generated
	19. Solid waste recycled (%)
	20. Solid Waste to Energy (%)
**Climate adaptation**	21. CO2 emission per capita
	22. Renewable energy % total
	23. Notre Dame Readiness Index
	24. IWRM (Integrated Water Resources Management)

B. SPECIFIC EXAMPLES FOR EACH OF THE INDICATORS

Category 1. Water Stress

Indicator 1: Water Scarcity

Principal: If a nation's extraction of water is greater than 20% of their water resource it is deemed to be unsustainable. The Water Exploitation Index, developed by Eurostat measures how sustainable a nation's water extraction is. The indicator presents I) the annual total fresh water abstraction in a country as a percentage of its long-term annual average (LTAA) available water from renewable fresh water resources; ii) the annual groundwater abstraction as a percentage of the country's long-term annual average groundwater available for abstraction; and iii) the annual surface water abstraction as a percentage of the country's long-term annual average surface water resources available for abstraction. The latter is calculated as the total fresh water resources (external inflow plus precipitation less evapotranspiration) less groundwater available for abstraction. The warning threshold of 20% for this indicator distinguishes a non-stressed from a water scarce region, with severe scarcity occurring where the WEI exceeds 40%. According to Eurostat the indicator has several limitations.

This index has been used in calculating indicator 1.

How to Calculate:

$$\boldsymbol{Indicator\ 1 = 10 - \frac{Water\ Exploitation\ Index}{10}}$$

If the Water Exploitation Index gives a low score, then the Indicator 1 value is a high as the nation is achieving the goal of sustainable water use.

Example: the water exploitation index (fresh surface and groundwater) for the Netherlands is 10.3.

Indicator 1 = 10- (10.3/10) = 8.97

Where to get the data

https://ec.europa.eu/eurostat/web/products-datasets/-/t2020_rd220

Accessed 28-03-2019

Indicator 2: Flood vulnerability

Principal:

Climate scenarios suggest that there is a risk or increased flooding in Europe in the next century This is due to increased intensity in the rainfall events, with longer dry periods(Dankers & Feyen, 2008). For this reason, vulnerability to flooding is included as a water stress. Flood vulnerability is measured from the susceptibility to floods over 26 years, between 1985 and 2011. This uses data based on flood occurrence so that countries that have invested in flood defense would have a lower vulnerability score due to fewer floods occurring. The goal is to have a flood vulnerability score of 9 for each nation.

How to Calculate

The Water Risk Atlas created by Aquaduct is used to identify flood vulnerable areas. The five colours on the map were each given a score between one and nine, where one is the highest level of flood risk and nine has the lowest level of flood risk. For those countries which have more than one colour we can calculate the weighted score.

Flood vulnerability Scores

Risk Category	Color Grade	Score

Extremely high risk	Dark Red	1
High risk	Red	3
Medium to high risk	Orange	5
Low to medium risk	Yellow	7
Low Risk	Cream	9

Example

Portugal lies within the orange for 80% of the country, and in the yellow for 20% in the north of the country. Thus, the score for Portugal becomes: 5*0.8 + 7*0.2 = 5.4

Therefore the overall score for Portugal is rounded to 5.

Data source https://www.wri.org/applications/maps/aqueduct-atlas/#x=34.19&y=45.27&s=ws!20!28!c&t=waterrisk&w=def&g=0&i=BWS-16!WSV-4!SV-2!HFO-4!DRO-4!STOR-8!GW-8!WRI-4!ECOS-2!MC-4!WCG-8!ECOV-2!&tr=ind-1!prj-1&l=4&b=terrain&m=group&init=y and go to the explore global water risk map, and the flood occurrence map.

Indicator 3: Transboundary cooperation

Principal: Many river basins, groundwater bodies and lakes are located at national borders or cross national borders. In order to successfully manage the water supply, there must be transboundary agreements in place(Orme et al., 2015). The indicator measures the presence of key international principles (Legal frameworks) for the transboundary basin. This indicator is taken from the Transboundary Water Assessment Program (TWAP) undertaken by UNEP. The TWAP Project has river level indicators, one of which is the transboundary legal frameworks available for each river basin. This is scored on a basin scale, as well as a score proportional to a countries land area within the basin. The proportional score is referred to as the Basin Country Unit (BCU) and has been used for this indicator. The goal for each BCU is to have good legal agreements in place, which would give an indicator score of 10.

How to Calculate

BCU score = 0-1 where 1 is high risk and 0 is low risk. If a country has multiple transboundary rivers, the arithmetic mean of the final score has been taken.

X = Mean of the legal framework indicator score

$$\mathbf{Indicator\ 3} = \mathbf{10} - (\mathbf{X} * \mathbf{10})$$

Example:

Go to the TWAP website, select the legal framework indicators. Download the legal framework data and use the normalized basin country unit scores. For example in the Netherlands, the BCU score for the Scheldt is 0.86 and for the Rhine is 0. The average BCU score is taken.

Relative Risk Category	Basin Country Unit Code	Country	River Basin	Area [000' km2]	Population [000']	Population-Area Weight	Score for BCU	Score for BCU (Normalized)	
4	SHLD_NLD	Netherland	Schelde	0	4	0.00	1.00	0.86	
1	RHIN_NLD	Netherland	Rhine	4	3418	0.05	7.00	0.00	
								0.428571	5.714285

$$\frac{0.86+0.00}{2} = 0.43 = X$$

$$\text{Indicator 3} = 10 - (0.43 * 10) = 5.71 \text{ or } 5.7$$

Data Source

http://twap-rivers.org/indicators/

Indicator 4: Tertiary Education Attainment

Principle: Attainment of education at a tertiary level yields highly educated professionals that are necessary in the creation or adoption of new technologies, fundamental for growth (Brunello et al., 2007) Without educated capital, the country would lack the skills required to cope with future challenges. A European target is for 40% of 30-34year olds to have tertiary education(European Comission, 2017)

How to Calculate:

A = % 25-64 year olds that have attained tertiary education

B = The lowest global tertiary education attainment, 2.29% (value of Eritrea)

40 has been taken as the maximum value so that the indicator score shows progression towards achieving the above goal.

$$\textbf{Indicator 4} = \frac{\mathbf{A - B}}{\mathbf{40} - \boldsymbol{B}} * \mathbf{10}$$

Example: For the Czech Republic, the value of A is 26.1.

$$\text{Indicator 4} = \frac{26.1 - 2.29}{40 - 26.1} * 10 = 6.3$$

Date: http://appsso.eurostat.ec.europa.eu/nui/show.do?dataset=edat_lfse_03&lang=eng

Category 2. Water Quality

The following Water Quality indicators are included to monitor the chemical, ecological surface water status and the chemical status of groundwater. Water sources can suffer from point and non-point source pollution which can lead to a degradation of ecosystem services and biodiversity loss (Bakker et al., 2012).

Indicator 5: Surface water Quality

Principal: The chemical quality of the surface water used as a drinking water source. A high score donates high water quality. The goal for each country is to ensure that every water source achieves good status or higher.

How to Calculate

$$\textbf{Indicator 5} = \frac{\textbf{surface water sources with a 'good' status}}{\textbf{total surface water sources assessed}} * \textbf{10}$$

Example:

The total surface water bodies in Spain (rivers and lakes only) that reached ‘good’ chemical status according to the wise water framework was 4225 out of 4716.

$$\text{Indicator 5} = \frac{4225}{4716} * 10 = 8.95$$

Data https://www.eea.europa.eu/data-and-maps/dashboards/wise-wfd

Last accessed 08-April 2019

Indicator 6: Ground water Quality

Principal: Measure of relative groundwater chemical quality. A lower Indicator score is given for poorer quality. The goal is for all groundwater sources to achieve good status.

How to Calculate

$$\textbf{Indicator 6} = \frac{\textbf{groundwater sources with a 'good' status}}{\textbf{total surface water sources assessed}} * \textbf{10}$$

Data: Groundwater Bodies Chemical status (GWB Chemical Status):

https://www.eea.europa.eu/data-and-maps/dashboards/wise-wfd

Last accessed 16th-January 2019

Example:

The total number of Swedish Groundwater bodies tested for their chemical quality was 40438. Of these 37955 achieved good chemical status.

$$\textbf{Indicator 6} = \frac{\textbf{37955}}{\textbf{40438}} * \textbf{10} = \textbf{9.4}$$

Giving Indicator 6 for Sweden as 9.4.

Indicator 7: Ecological Water Quality

Principal: The quality of the surface water used as a drinking water source. A high score donates high water quality. The goal is for each surface water source to achieve ‘good’ ecological status.

How to Calculate

$$\textbf{Indicator 7} = \frac{\textbf{surface water sources with a "good/high}'\textbf{ecological status}}{\textbf{total surface water sources assessed}} *\textbf{10}$$

Example: For the Spain, a total 2584 lakes and rivers gained a good or high Ecological status, of a total of 4716 surface water bodies.

$$\textbf{Indicator 7} = \frac{\textbf{2584}}{\textbf{4716}} * \textbf{10} = \textbf{5.5}$$

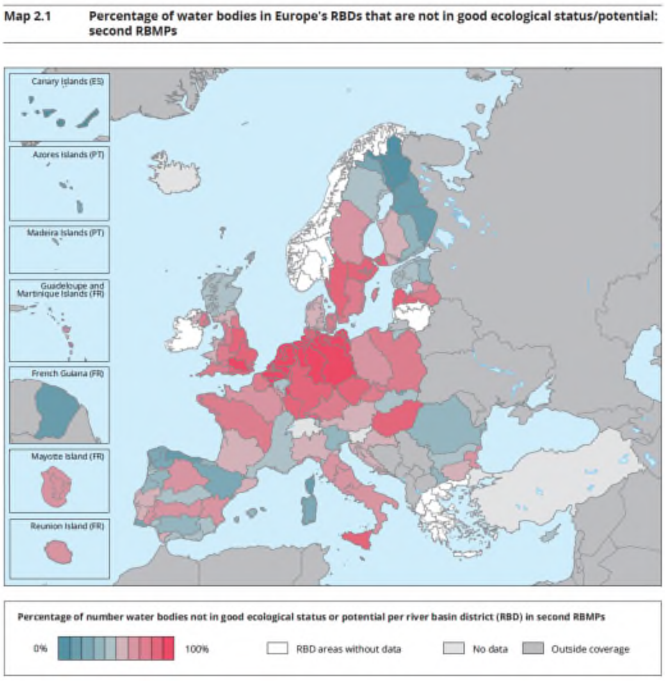

Where to get the data

https://www.eea.europa.eu/data-and-maps/figures/proportion-of-classified-surface-water-5 last accessed 16th January 2019. Go to Surface Water Bodies (SWB) ecological status

Category 3. Access basic water services

Indicator 8: Drinking Water Quality

Principal: The quality of the water supply gives an indication of the capacity of the system for filtration and transport(Hoekstra et al., 2018).This is taken from the level of compliance with drinking water regulations, for EU countries this is the Drinking Water Directive. The goal is for the compliance value to be 100.

How to Calculate

$$\textbf{Indicator 8} = \frac{\textbf{Level of compliance idicator value}}{\textbf{10}}$$

Example: the compliance rate for Poland has a value of 99.8, giving a value of 9.98 for indicator 8.

Where to get the data

Use Table 1 on pages 12/13:

http://ec.europa.eu/environment/water/water-drink/pdf/reports/EN.pdf

Indicator 9: Population connected to a drinking water supply

Principal: This represents the percentage of the population that receives a piped drinking water supply rather than reliant on a local spring source or an alternative source. This shows the development of water infrastructure present (Hoekstra et al., 2018). A lower indicator score is given when the percentage is low. The goal is for 100% of the population to receive a piped source of drinking water.

How to Calculate

A = Population connected to a piped drinking water supply

B = Total population

$$\textbf{Indicator 9} = \frac{\textbf{A}}{\textbf{B}} * \textbf{10}$$

Example: In Poland, 91.8% of the population is connected to a drinking water supply. This gives an indicator value of 9.2.

Where to get the data

https://washdata.org/data

Indicator 10: Percentage of the population connected to improved sanitation

Principal: A measure of the percentage of the population covered by wastewater collection and treatment. Improved sanitation facilities mean that excreta has been separated away from human, this does not distinguish between separation techniques. Separation can include via a sewerage network, in-situ treatment and disposal as well as onsite storage with removal for treatment(JMP, 2017). A lower Indicator score is given where the percentage is lower. The goal is for 100% of the population to have improved sanitation.

How to Calculate

A = The percentage of the population connected to improved sanitation.

$$\textbf{Indicator 10} = \frac{\textbf{A}}{\textbf{10}}$$

Example: In Sweden, 92 % of the population is connected to improved sanitation, this gives an indicator value of 9.2.

Where to get the data

https://washdata.org/data.

Use household data, go the world file. Use the most recent data (e.g. 2015). Safely managed sanitation at the national level.

Indicator 11: Water affordability

Principal: Within Europe, water is readily available, usually piped directly into the home. However, some individuals face financial difficulties in being able to afford this water. If water costs more than 3% of the individuals income, then they are said to be water poor (García-Valiñas et al., 2010). The goal is to ensure that whilst water is used sustainably, it remains affordable with the pricing below 4% of the average income.

How to calculate

A: average water bill per month

B: average income per month

0.3 is the conversion value to give an indicator score between 0-10.

$$\textbf{Indicator 11} = 10 - \left(\frac{A}{B} * \frac{1}{0.39} * 100\right)$$

Example: In Spain

A = 13.65(US $)

B = 2265 (US $)

$$\textbf{Indicator 11} = 10 - \left(\frac{13.65}{2265} * \frac{1}{0.39} * 100\right) = 8.5$$

Where to get the data

Water bill per month (USD): http://cdn.thejournal.ie/media/2016/09/tariffs-4.jpg last accessed 20th December 2018

Average monthly income (USD): https://www.worlddata.info/average-income.php last accessed 8 April 2019.

Category 4. Infrastructure

Indicator 12: Infrastructure investment

Principal: Investment into infrastructure is required for the infrastructure to be properly maintained and for new technologies to be implemented. The goal for infrastructure investment is 3.8% of national GDP(Santarsiero et al., 2016).

How to Calculate:

To calculate this, the most recent data on infrastructure investment is used. This is from 2010. The GDP of 2010 is then also used to measure the amount of investment relative to the GDP. In order to create a score from 0-10, this is then multiplied by a conversion factor of 263.

A= 2010 infrastructure investment

B = 2010 GDP

$$\textbf{Indicator 12} = \frac{\boldsymbol{A}}{\boldsymbol{B}} * \mathbf{263}$$

Example

For Poland, A = 722453066 and B= 360344273490, giving the infrastructure investment as 2% of GDP and an indicator score of 5.28.

$$= \frac{\mathbf{722453066}}{\mathbf{360344273490}} * \mathbf{263} = \mathbf{5.28}$$

Where to get the data

Transport infrastructure investment and maintenance spending (OECD):

https://stats.oecd.org/Index.aspx?DataSetCode=ITF_INV-MTN_DATA

Infrastructure investment is found in euros and converted to US$ using the average 2010 exchange rate.

World Bank GDP (current USD): https://data.worldbank.org/indicator/ny.gdp.mktp.cd

Indicator 13: Water leakage

Principal: Leakages from the water network effects water use efficiency as well as water quality(EEA, 2001). Target 6.4 of the SDGs is to increase water-use efficiency, therefore the goal is for no water to be lost due to leakage.

How to Calculate

Leakage rates of 50% are taken to be the maximum value and would thus score 0.

$$\textbf{Indicator 13} = \mathbf{10} - \frac{\textbf{percentage water lost}}{\mathbf{10}} * \mathbf{2}$$

Example: In Sweden, distribution losses account for 18% of water lost in the system.

$$\textbf{Indicator 13} = 10 - \left(\frac{18}{10} * 2\right) = 6.4$$

Where to get the data

Percent water lost per kilometer of network:

https://www.danva.dk/media/3645/eureau_water_in_figures.pdf (EU) *illustrated in Figure 19 of the report, shown in the graph below.*

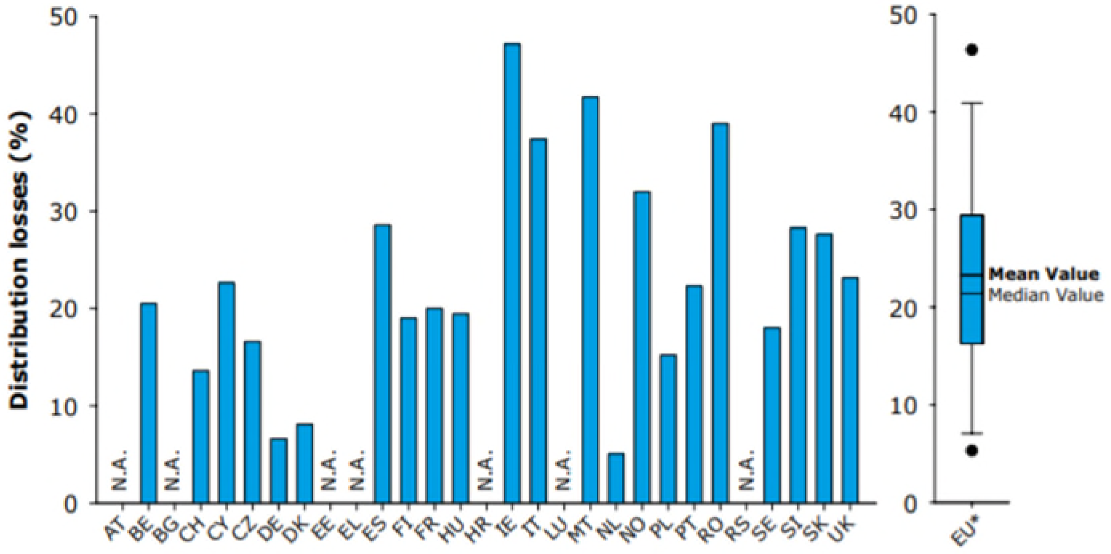

Category 5. Waste water treatment

Indicator 14: Secondary Waste Water Treatment (WWT)

Principal: Measure of the population connected to secondary wastewater treatment plants. Primary treatment treats the physical water contamination, whereas secondary water treatment also treats the biological and organic contamination(de Moel et al., 2006). The goal is for all nations to have 100% Secondary WWT.

How to Calculate

A = Percentage of secondary WWT

B = Percentage of tertiary WWT

$$\textbf{Indicator 14} = \frac{\mathbf{A + B}}{\mathbf{10}}$$

Example: In Sweden the percentage of secondary WWT is 2.5%, the percentage of tertiary WWT is 92.9%. Therefore, the amount of waste that is treated to at least a secondary level is 95.4%.

$$\textbf{Indicator 14} = \frac{\mathbf{2.5 + 92.9}}{\mathbf{10}} = \mathbf{9.54}$$

Definition of secondary WWT: Definition secondary WWT: Secondary treatment: process generally involving biological treatment with a secondary settlement or other process, with a BOD removal of at least 70% and a COD removal of at least 75% (OECD, 2013).

Where to get the data

https://www.eea.europa.eu/data-and-maps/indicators/urban-waste-water-treatment/urban-waste-water-treatment-assessment-4 Click on the Table and data are shown for secondary and tertiary treatment

Indicator 15: Percentage of tertiary Waste Water Treatment

Principal: Measure of the population connected to tertiary wastewater treatment plants. Tertiary wastewater treatment is the final stage in the treatment process and removes inorganic compounds, carbonaceous matter and additional solids before the water is released into surface water. Reuse of water reduces required water extraction(Cirelli & Salgot, 2001). The goal is for all nations to have the highest level of wastewater treatment, 100% being tertiary treatment.

How to Calculate

$$\textbf{Indicator 15} = \frac{\textbf{Percentage of tertiary treatment}}{\mathbf{10}}$$

Example

As seen above, 92.9% of Sweden's water treatment is to a tertiary level.

$$\textbf{Indicator 15} = \frac{\mathbf{92.9}}{\mathbf{10}} = \mathbf{9.3}$$

Definition of tertiary: Tertiary treatment: treatment of nitrogen and/or phosphorous and/or any other pollutant affecting the quality or a specific use of water (microbiological pollution, color, etc.). (OECD, 2013)

Where to get the data

https://www.eea.europa.eu/data-and-maps/indicators/urban-waste-water-treatment/urban-waste-water-treatment-assessment-4 Click on the Table and data are shown for secondary and tertiary treatment

Indicator 16: Nutrient Recovery (%)

Principle: Nutrients recovered from sewage sludge by use as agricultural manure or via composting. In order to fully implement the circular economy all nutrients should be recovered from wastewater (Jurgilevich et al., 2016).

How to calculate

A. Dry weight of sludge produced in wastewater treatment plants serving the city
B. Dry weight of sludge going to landfill

C. Dry weight of sludge thermally processed
D. Dry weight of sludge disposed in agriculture

E. Dry weight of sludge disposed by composting

F. Dry weight of sludge disposed by other means

(As a check, A should equal B + C + D + E + F)

$$\textbf{Indicator 16} = \frac{D+E}{A} * \frac{\%\ \text{secondary WWT coverage}}{100} * 10$$

To measure the full potential of nutrient and energy recovery from wastewater by using wastewater abstracted sewage sludge, secondary WWT is incorporated in the calculation and not primary WWT. In this way the concept of urban metabolism is better represented. Secondary WWT produces more sewage sludge than Primary WWT.

Example: German sewage sludge is used both for agriculture and in compost.

With 491 thousand tons in Agriculture and 264 thousand tons in compost and a secondary WWT of 94.4%.

$$\frac{491 + 264}{1795} \times \frac{95.4}{100} \times 10 = 4.01$$

This gives the Indicator 16 value of 4.0 for Germany.

Where to find the data

https://data.europa.eu/euodp/data/dataset/hzWkcfKt5mxEaFijeoA

Go to visualize Table and select the data for the calculations

Indicator 17: Waste Water to energy

Principle: Energy can be gained from sewage waste by incineration(van der Hoek et al., 2017). To achieve the goal of the circular economy, energy needs to be gained from wastewater.

How to calculate

A. Dry weight of sludge produced in wastewater treatment plants serving the city
B. Dry weight of sludge going to landfill
C. Dry weight of sludge thermally processed
D. Dry weight of sludge disposed in agriculture

E. Dry weight of sludge disposed by composting

F. Dry weight of sludge disposed by other means

(As a check, A should equal B + C + D + E + F)

$$\textbf{Indicator 17} = \frac{C}{A} * \frac{\%\ \text{secondary WWT coverage}}{100} * 10$$

Example: The Netherlands incinerates 6608 thousand tons of waste, of a total of 9497 thousand tons.

$$\frac{6608}{9497} \times \frac{99.4}{100} \times 10 = 6.9$$

This gives a value of 6.9 for indicator 16 for the Netherlands.

Where to find the data

https://data.europa.eu/euodp/data/dataset/hzWkcfKt5mxEaFijeoA

Go to visualize Table and select the data for the calculations

Category 6. Solid Waste treatment

Indicator 18: Municipal Solid Waste (MSW) collected

Principal: Solid waste collected by the municipality, not including large industrial waste. Untreated municipal waste can result in pollution and the release of toxins into the environment, there is a link between the quantity of waste produced and the presence of garbage in water bodies (Hoekstra et al., 2018). The goal is to reduce the solid waste produced to 10% of 2010 levels by 2010 based on the Spanish waste prevention objectives (EEA, 2014).

How to Calculate

$$\textbf{Indicator 18} = \frac{\textbf{percent total waste collected}}{\textbf{10}}$$

Example: In the United Kingdom, 100% of solid waste is collected, giving an indicator value of 10.

Where to get the data

http://data.un.org/Data.aspx?q=municipal+waste&d=ENV&f=variableID%3a1814

Indicator 19: Municipal Solid Waste recycled

Rationale: Percentage of solid waste that is recycled or composted. To ensure that there is a reduced loss of raw materials, waste should be recycled. The EU recycling target for 2030 is that 65% of municipal waste is recycled.

How to Calculate

A = Percent of municipal solid waste that is recycled

$$\textbf{Indicator 19} = \frac{\boldsymbol{A}}{\textbf{65}} \times \textbf{10}$$

Example: In Greece, 16.4% of solid waste is recycled, giving an indicator value of 2.5.

$$\textbf{Indicator 19} = \frac{\textbf{16.4}}{\textbf{65}} \times \textbf{10} = \textbf{2.52}$$

Data: https://stats.oecd.org/ Go to Environment and click on waste and then to municipal waste.

Indicator 20: Solid waste energy recovery

Principal: Percentage of solid waste that is incinerated with energy recovery. (Environmental Service Association, 2018). In the ideal scenario, 100% of energy available for recovery should be recovered.

How to calculate

This indicator represents the percentage of the total collected municipal waste that incinerated with energy recovery (techniques). However, when solid waste is recycled or composted, it is not possible to also use it for incineration with energy recovery, while both practices are sustainable. Therefore the % solid waste that is recycled or composted is subtracted from the total (100%) of collected municipal waste to obtain the potential percentage of solid waste that can be incinerated with energy recovery (in numerator). Thus this indicator is calculated as shown below.

A: municipal solid waste incinerated with energy recovery

B: municipal solid waste recycled

C: municipal solid waste generated

$$\textbf{Indicator 20} = \frac{\text{A}}{\text{C} - \text{B}} \times 10$$

Example: The municipal solid waste incinerated with energy recovery was 10863 thousand tons in Poland, 2,867 thousand tons of this was recycled and 1318 thousand tons was incinerated with energy recovery.

$$\textbf{Indicator 20} = \frac{1318}{(10863-2867)} \times 10 = 1.7$$

Giving 1.7 as the indicator 20 value for Poland.

Where to get the data

https://stats.oecd.org/ last accessed 9th January 2019. Go to Environment and click on waste and then to municipal waste.

Category 7. Climate adaptation

Indicator 21: CO2 emissions

Principal: CO2 emissions are a greenhouse gas (GHG) and so increase the global warming effect. Maintaining high GHG emissions will have a negative impact on the climate and resources(van Vuuren et al., 2011). Without monitoring CO2 emissions alternative freshwater sources (such as decarbonization) increase index scores with their negative environmental impact being unaccounted for (Dawoud & Al Mulla, 2012). Reducing greenhouse gas emissions by 40%(from 1990 levels) is a key target in the 2030 climate and energy framework(European Council, 2014).

How to calculate

A= 1990 CO_2 emissions per capita

B= 2014 CO_2 emissions per capita

The target of 40% reduction is used.

$$\textbf{Indicator 21} = \frac{A-B}{A\times 0.4}*10$$

Example:

In the example of Belgium. The CO_2 emissions per capita in 1990 were 10.64 metric tons per capita and had decreased to 8.33 metric tons per capita in 2014.

$$\textbf{Indicator 21} = \frac{10.64-8.33}{10.64\times 0.4}*10 = 5.43$$

Where to get the data

https://data.worldbank.org/indicator/EN.ATM.CO2E.PC

Indicator 22: Renewable energy share (%) of total final energy consumption

Principal: Increasing renewable energy sources is a way to mitigate climate change, and move away from a fossil resource based economy (Owusu & Asumadu-Sarkodie, 2016). A key target for the climate and energy framework 2030 for the EU is to have a share of 27% renewable energy for the countries energy provision (European Comission, 2016) (Article 3 of Directive 2008/98/EC).

How to calculate

$$\textbf{Indicator 22}: \frac{\textbf{Renewable energy share } (\%)}{\textbf{27}} * \textbf{10}$$

Example:

Using the Netherlands as an example, in 2015 5.89% of the energy was from a renewable source. This gives an indicator score of 2.18.

$$\frac{\textbf{5.89 } (\%)}{\textbf{27}} * \textbf{10} = \textbf{2.18}$$

Where to get the data

http://databank.worldbank.org/data/reports.aspx?source=1261&series=2.1_SHARE.TOTAL.RE.IN.TFEC

Indicator 23: Notre Dame readiness index

Principal: Notre Dame Global Adaptation Initiative (ND-GAIN) measures overall readiness by considering three components – economic readiness, governance readiness and social readiness. Readiness measures a country's

ability to leverage investments and convert them to adaptation actions. (Chen, 2015). The goal is to have a score of 1 in readiness.

How to calculate

The ND-GAIN score is given as a value between 0-100 for each country with high values showing more readiness to adapt to climate change impacts.

$$\textbf{Indicator 23} = \textbf{ND} - \textbf{GAIN Score} \times \textbf{10}$$

Example

Using Ireland as an example, the ND-GAIN readiness score is 0.640, giving an indicator score of 6.4.

$$\mathbf{0.640 \times 10 = 6.4}$$

Where to get the data

https://gain-new.crc.nd.edu/ranking/readiness

Indicator 24: Integrated Water Resources Management Implementation

Principal: There are many synergies between water management and the development of sanitation, agriculture and energy, however water targets for these industries are lacking in their respective goals. Including an IWRM indicator shows the degree to which an integrated approach is being used (Ait-Kadi, 2016)**.**The indicator measures the percentage of Integrated Water Resources Management(IWRM) in river basin management plans. The goal is for all basins to be managed using IWRM.

How to Calculate:

$$\textbf{Indicator 24} = \frac{\boldsymbol{IWRM\ implementation}}{\mathbf{10}}$$

Example

Poland has 40% IWRM implementation, therefore the indicator value is 4.

Where to get the data

http://www.sdg.org/datasets/406e085811164632b16f701eecdbdefd_0

Click on data

Indicator identification references

Ait-Kadi, M. (2016). Water for Development and Development for Water: Realizing the Sustainable

Development Goals (SDGs) Vision. *Aquatic Procedia*, *6*, 106–110. https://doi.org/10.1016/j.aqpro.2016.06.013
Allen, C., Metternicht, G., & Wiedmann, T. (2018). Initial progress in implementing the Sustainable Development Goals (SDGs): a review of evidence from countries Assessing absolute sustainability of global cities View project The Arab Sustainable Development Report, First Edition (ESCWA, UNEP) View project Initial progress in implementing the Sustainable Development Goals (SDGs)-A review of evidence from countries. https://doi.org/10.1007/s11625-018-0572-3
Arup. (2014). CITY RESILIENCE INDEX.
Asian Development Bank. (2016). *ASIAN WATER DEVELOPMENT STRENGTHENING WATER SECURITY IN ASIA AND THE PACIFIC*. Retrieved from www.adb.org
Bakker, K., Kolb//, K. J., & Commons, W. (2012). Water Security: Research Challenges and Opportunities, *337*. https://doi.org/10.1126/science.1226337
Barnett, P. (2015). *IF WHAT GETS MEASURED GETS MANAGED, MEASURING THE WRONG THING MATTERS*. Retrieved from http://www.strategicmanagementforum.org.
Batten, J. (2016). *Sustainable Cities Water Index*. Retrieved from https://www.arcadis.com/media/4/6/2/%7B462EFA0A-4278-49DF-9943-C067182CA682%7DArcadis_Sustainable_Cities_Water_Index-Web.pdf
Bhaduri, A., Bogardi, J., Siddiqi, A., Voigt, H., Vörösmarty, C., Pahl-Wostl, C., … Osuna, V. R. (2016). Achieving Sustainable Development Goals from a Water Perspective. *Frontiers in Environmental Science*, *4*, 64. https://doi.org/10.3389/fenvs.2016.00064
Brundtland, G. H. (1987). Our Common Future: Report of the World Commission on Environment and Development. *United Nations Commission*, *4*(1), 300. https://doi.org/10.1080/07488008808408783
Brunello, G., Garibaldi, P., & Wasmer, E. (2007). Higher Education, Innovation and Growth. In *Education and Training in Europe* (pp. 56–70). Oxford University Press. https://doi.org/10.1093/acprof:oso/9780199210978.003.0004
Chen, C. ; (2015). *University of Notre Dame Global Adaptation Index Country Index Technical Report*. Retrieved from http://index.gain.org/about/reference.
Cirelli, G., & Salgot, M. (2001). Role of water reuse for enhancing integrated water management in Europe and Mediterranean countries. https://doi.org/10.2166/wst.2001.0571
Dankers, R., & Feyen, L. (2008). Climate change impact on flood hazard in Europe : An assessment based on high-resolution climate simulations, *113*(December 2007), 1–17. https://doi.org/10.1029/2007JD009719
Dawoud, M. A., & Al Mulla, M. M. (2012). *Environmental Impacts of Seawater Desalination: Arabian Gulf Case Study*. *International Journal of Environment and Sustainability* (Vol. 1). Retrieved from www.sciencetarget.com
de Moel, P. J., Verberk, J. Q. J. C., & van Dijk, J. C. (2006). *Drinking Water: Principles and Practices* (first edit). Delft: World Scietific Publishing Co.
de Vries, B. J. M. (2012). *Sustainability Science*. Cambridge: Cambridge University Press. https://doi.org/10.1017/CBO9780511794469
Dickens, C., Smakhtin, V., McCartney, M., O'Brien, G., & Dahir, L. (2019). Defining and Quantifying National-Level Targets, Indicators and Benchmarks for Management of Natural Resources to Achieve the Sustainable Development Goals. *Sustainability*, *11*(2), 462. https://doi.org/10.3390/su11020462
EEA. (2001). Indicator Fact Sheet Signals 2001 – Soil, 1–5.
EEA. (2014). Waste prevention objectives. Retrieved April 10, 2019, from https://scp.eionet.europa.eu/facts/WPP/objectives
EEA. (2018). Water use by sectors — European Environment Agency. Retrieved August 28, 2018, from https://www.eea.europa.eu/themes/water/water-resources/water-use-by-sectors
Environmental Service Association. (2018). Energy for the Circular Economy: an overview of Energy from Waste in the UK.
Equal Measures 2030. (2019). *HARNESSING THE POWER OF DATA FOR GENDER EQUALITY Introducing the 2019 EM2030 SDG Gender Index*. Retrieved from https://data.em2030.org/wp-content/uploads/2019/05/EM2030_2019_Global_Report_ENG.pdf
EurEau. (2017). *Water in Figures*. Retrieved from https://www.danva.dk/media/3645/eureau_water_in_figures.pdf
European Comission. (2015). Science for Environment Policy: Indicators for Sustainable Cities, (12). https://doi.org/10.2779/61700
European Comission. Proposal for a DIRECTIVE OF THE EUROPEAN PARLIAMENT AND OF THE COUNCIL on the promotion of the use of energy from renewable sources (recast) (2016). Retrieved from https://eur-lex.europa.eu/legal-content/EN/TXT/?uri=CELEX:52016PC0767R%2801%29
European Comission. (2017). *Europe 2020 Targets*. Retrieved from https://ec.europa.eu/eurostat/documents/4411192/4411431/Europe_2020_Targets.pdf

European Commission. (2017). *Indicators to measure Social Protection Performance*. https://doi.org/10.2841/158380

European Council. (2014). *2030 Climate and Energy Policy Framework*. Retrieved from http://data.consilium.europa.eu/doc/document/ST-169-2014-INIT/en/pdf

Gain, A. K., Giupponi, C., & Wada, Y. (2016). Measuring global water security towards sustainable development goals. *Environmental Research Letters*, *11*(12), 124015. https://doi.org/10.1088/1748-9326/11/12/124015

García-Valiñas, M. D. L. Á., Martínez-Espiñeira, R., & González-Gómez, F. (2010). Measuring water affordability: A proposal for urban centres in developed countries. *International Journal of Water Resources Development*, *26*(3), 441–458. https://doi.org/10.1080/07900627.2010.491971

Gawlik, B., Easton, P., Koop, S., Van Leeuwen, K., & Elelman, R. (2017). *Urban Water Atlas for Europe*.

Georgeson, L., & Maslin, M. (2018). Putting the United Nations Sustainable Development Goals into practice: A review of implementation, monitoring, and finance. https://doi.org/10.1002/geo2.49

Giordano, M., & Shah, T. (2014). From IWRM back to integrated water resources management. *International Journal of Water Resources Development*, *30*(3), 364–376. https://doi.org/10.1080/07900627.2013.851521

Guppy, L., Mehta, P., & Qadir, M. (2019). Sustainable development goal 6: two gaps in the race for indicators. *Sustainability Science*, *14*(2), 501–513. https://doi.org/10.1007/s11625-018-0649-z

HLPW. (2018). Making Every Drop Count, (March).

Hoekstra, A. Y., Buurman, J., & Van Ginkel, K. C. H. (2018). Urban water security: A review E-mail: a.y.hoekstra@utwente.nl. *Environ. Res. Lett*, *13*, 53002. https://doi.org/10.1088/1748-9326/aaba52

Hoekstra, A. Y., Mekonnen, M. M., Chapagain, A. K., Mathews, R. E., & Richter, B. D. (2012). Global Monthly Water Scarcity: Blue Water Footprints versus Blue Water Availability. *PLoS ONE*, *7*(2), e32688. https://doi.org/10.1371/journal.pone.0032688

Hunter, P. R., MacDonald, A. M., & Carter, R. C. (2010). Water Supply and Health. *PLoS Medicine*, *7*(11), e1000361. https://doi.org/10.1371/journal.pmed.1000361

JMP. (2017). Sanitation. Retrieved August 2, 2018, from https://washdata.org/monitoring/sanitation

Jurgilevich, A., Birge, T., Kentala-Lehtonen, J., Korhonen-Kurki, K., Pietikäinen, J., Saikku, L., & Schösler, H. (2016). Transition towards circular economy in the food system. *Sustainability (Switzerland)*, *8*(1), 1–14. https://doi.org/10.3390/su8010069

Kaddoura, S., & El Khatib, S. (2017). Review of water-energy-food Nexus tools to improve the Nexus modelling approach for integrated policy making. *Environmental Science and Policy*, *77*(August), 114–121. https://doi.org/10.1016/j.envsci.2017.07.007

Koop, S. H. A., & van Leeuwen, C. J. (2015a). Application of the Improved City Blueprint Framework in 45 Municipalities and Regions. *Water Resources Management*, *29*(13), 4629–4647. https://doi.org/10.1007/s11269-015-1079-7

Koop, S. H. A., & van Leeuwen, C. J. (2015b). Assessment of the Sustainability of Water Resources Management: A Critical Review of the City Blueprint Approach. *Water Resources Management*, *29*(15), 5649–5670. https://doi.org/10.1007/s11269-015-1139-z

Kurian, M. (2017). The water-energy-food nexus: Trade-offs, thresholds and transdisciplinary approaches to sustainable development. *Environmental Science and Policy*, *68*, 97–106. https://doi.org/10.1016/j.envsci.2016.11.006

Lambin, E. F., & Meyfroidt, P. (2011). Global land use change, economic globalization, and the looming land scarcity. *Proceedings of the National Academy of Sciences of the United States of America*, *108*(9), 3465–3472. https://doi.org/10.1073/pnas.1100480108

Malik, O. A., Hsu, A., Johnson, L. A., & de Sherbinin, A. (2015). A global indicator of wastewater treatment to inform the Sustainable Development Goals (SDGs). *Environmental Science & Policy*, *48*, 172–185. https://doi.org/10.1016/J.ENVSCI.2015.01.005

Medema, W., McIntosh, B. S., & Jeffrey, P. J. (2008). From Premise to Practice: a Critical Assessment of Integrated Water Resources Management and Adaptive Management Approaches in the Water Sector. *Ecology and Society*, *13*(2), art29. https://doi.org/10.5751/ES-02611-130229

Niasse, M. (2006). *Transboundary River Basins. Atlas on Regional Integration in West Africa* (Vol. 3).

Nilsson, M., Griggs, D., & Visbeck, M. (2016). Map the Interactions between Sustainable Development Goals. *Proc. Nat. Acad. Sci*, *382*, 5125–5129. https://doi.org/10.1787/agr_outlook-2015-en

OECD. (2017). *Measuring Distance to SDG Targets*. Retrieved from http://www.oecd.org/sdd/OECD-Measuring-Distance-to-SDG-Targets.pdf

Orme, M., Cuthbert, Z., Sindico, F., & Gibson, J. (2015). Good Transboundary Water Governance in the 2015 Sustainable Development Goals: A Legal Perspective. *University of Strathclyde*. https://doi.org/10.1080/02508060.2015.1099083

Owusu, P. A., & Asumadu-Sarkodie, S. (2016). A review of renewable energy sources, sustainability issues and climate change mitigation. *Cogent Engineering*, *3*(1). https://doi.org/10.1080/23311916.2016.1167990

Petit, O. (2016). Paradise lost? The difficulties in defining and monitoring Integrated Water Resources Management indicators. *Current Opinion in Environmental Sustainability*, *21*(21), 58–64. https://doi.org/10.1016/j.cosust.2016.11.006

Pires, A., Morato, J., Peixoto, H., Botero, V., Zuluaga, L., & Figueroa, A. (2017). Sustainability Assessment of indicators for integrated water resources management. *Science of The Total Environment*, *578*, 139–147. https://doi.org/10.1016/J.SCITOTENV.2016.10.217

Policy Research Initiative. (2007). Canadian Water Sustainability Index (CWSI). Project Report. https://doi.org/10.2166/washdev.2012.005

Pradhan, P., Costa, L., Rybski, D., Lucht, W., & Kropp, J. P. (2017). A Systematic Study of Sustainable Development Goal (SDG) Interactions Earth ' s Future. *Earth's Future*, 1169–1179. https://doi.org/10.1002/eft2.266

Rasul, G. (2016). Managing the food, water, and energy nexus for achieving the Sustainable Development Goals in South Asia. *Environmental Development*, *18*, 14–25. https://doi.org/10.1016/J.ENVDEV.2015.12.001

Reidhead, W. (UN-W., Harlin, J. (UNEP), Alabaster, G. (UN-H., Slaymaker, T. (UNICEF), Frenken, K. (FAO), Arduino, G. (UNESCO), … Abrate, T. (WMO). (2016). Monitoring Water and Sanitation in the 2030 Agenda for Sustainable Development - An Introduction, 1–12.

Robinson, J. B., & Robinson, J. (2004). Squaring the Circle? Some Thoughts on the Idea of Sustainable Development. https://doi.org/10.1016/j.ecolecon.2003.10.017

Santarsiero, G., Chiauzzi, L., & Masi, A. (2016). Bridging Global Infrastructure Gaps, (September). https://doi.org/10.13140/RG.2.2.26758.37440

Savenije, H. H. G., & Van der Zaag, P. (2008). Integrated water resources management: Concepts and issues. *Physics and Chemistry of the Earth, Parts A/B/C*, *33*(5), 290–297. https://doi.org/10.1016/J.PCE.2008.02.003

Scherer, L., Behrens, P., de Koning, A., Heijungs, R., Sprecher, B., & Tukker, A. (2018). Trade-offs between social and environmental Sustainable Development Goals. *Environmental Science and Policy, 90*. https://doi.org/10.1016/j.envsci.2018.10.002

SDEWES. (2017). *SDEWES Index. Plenary Lecture at 12 th SDEWES Conference*. Retrieved from https://www.sdewes.org/SDEWES Index Atlas.pdf

Siemens AG. (2012). *The Green City Index*. Retrieved from http://etms.espon.eu/rankings/2012_European_Green_City_Index_sum_report.pdf

UN. (2015). *World Population Prospects The 2015 Revision*. Retrieved from https://esa.un.org/unpd/wpp/Publications/Files/Key_Findings_WPP_2015.pdf

United Nations. (2015a). *The Millenium Development Report 2015*. Retrieved from https://www.un.org/millenniumgoals/2015_MDG_Report/pdf/MDG 2015 rev (July 1).pdf

United Nations. (2015b). Transforming our world: the 2030 Agenda for Sustainable Development. *General Assembley 70 Session*, *16301*(October), 1–35. https://doi.org/10.1007/s13398-014-0173-7.2

United Nations. (2018). *Sustainable Development Goal 6 Synthesis Report on Water and Sanitation*.

United Nations Department of Economic and Social Affairs. (2013). Scenarios for Sustainable Development for Rio+20, (February).

van der Hoek, J. P., Struker, A., & de Danschutter, J. E. M. (2017). Amsterdam as a sustainable European metropolis: integration of water, energy and material flows. *Urban Water Journal*, *14*(1), 61–68. https://doi.org/10.1080/1573062X.2015.1076858

van Vuuren, D. P., Edmonds, J., Kainuma, M., Riahi, K., Thomson, A., Hibbard, K., … Rose, S. K. (2011). The representative concentration pathways: an overview. *Climatic Change*, *109*(1–2), 5–31. https://doi.org/10.1007/s10584-011-0148-z

Water Research Foundation. (2016). Residential End Uses of Water Study(2016. 1999). Retrieved June 4, 2019, from http://www.allianceforwaterefficiency.org/residential-end-uses-of-water-study-1999.aspx

World Bank. (2018). *Atlas of Sustainable Development Goals 2018: From World Development Indicators. World Bank publication*. https://doi.org/10.1596/978-1-4648-1250-7

ANNEX 2 RESULTS OF SOME OF THE INDICATOR SCORES FOR THE 28 EU MEMBER STATES

Annex 2 provides the scores for some of the key indicators provided in Table 1 in Annex 1 of this Supplementary Material

1.Water Scarcity

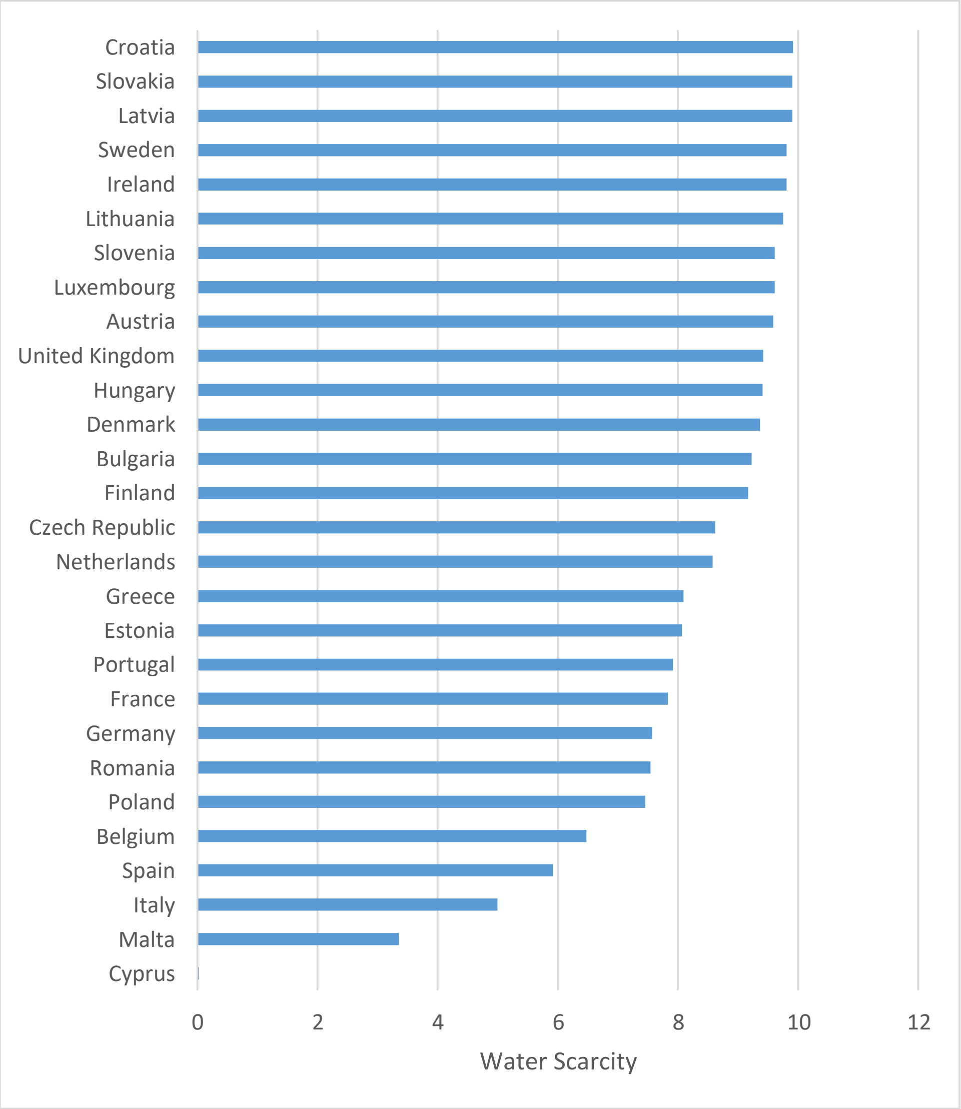

4. Surface water quality

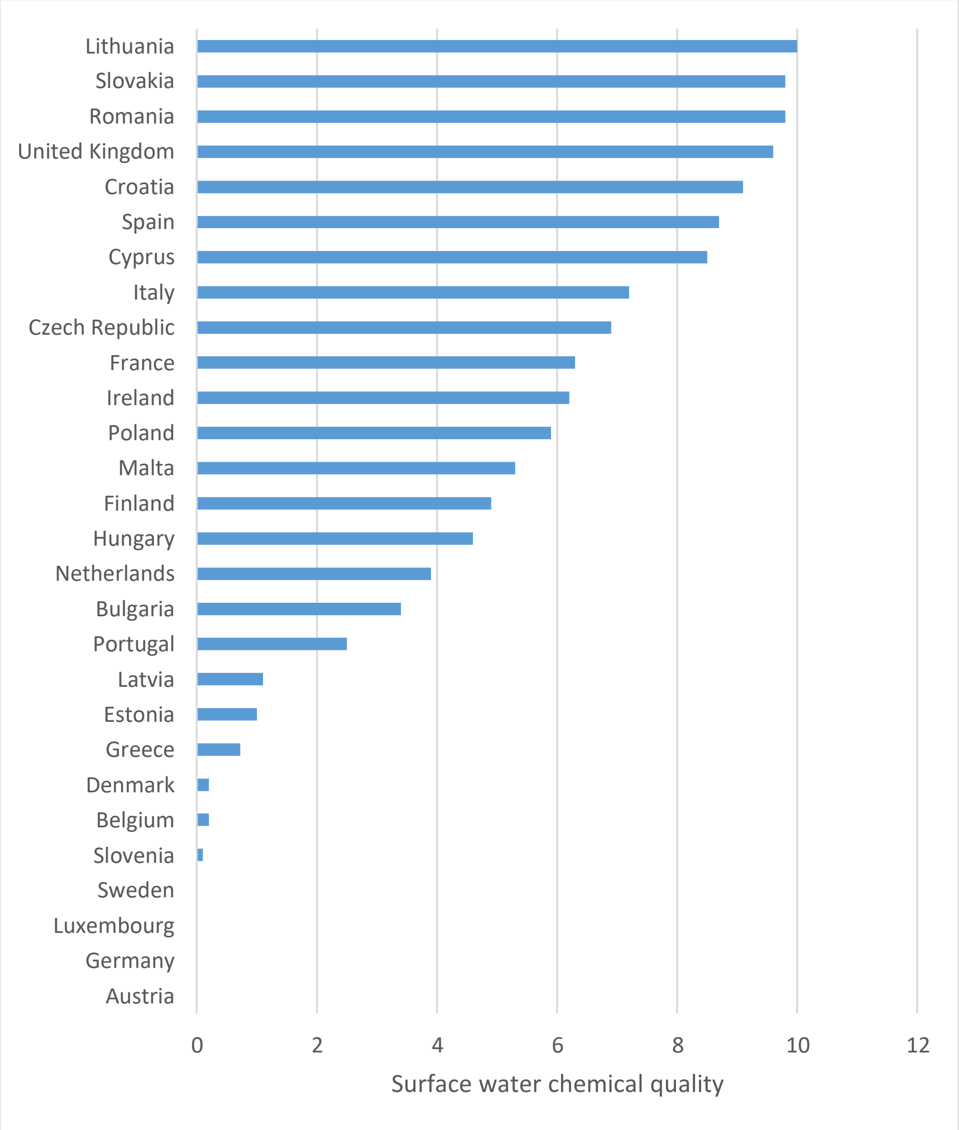

5. Groundwater quality

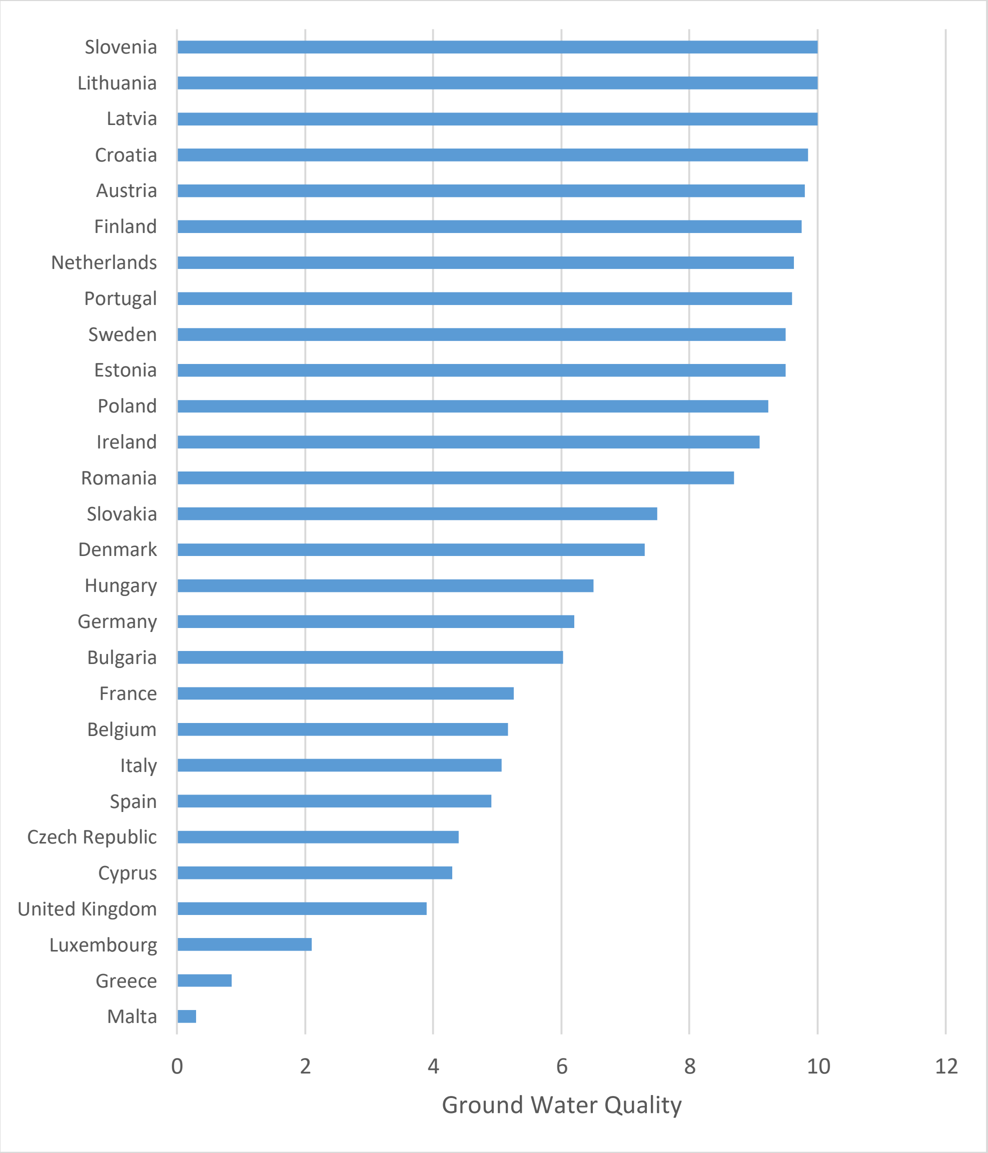

6. Ecological water quality

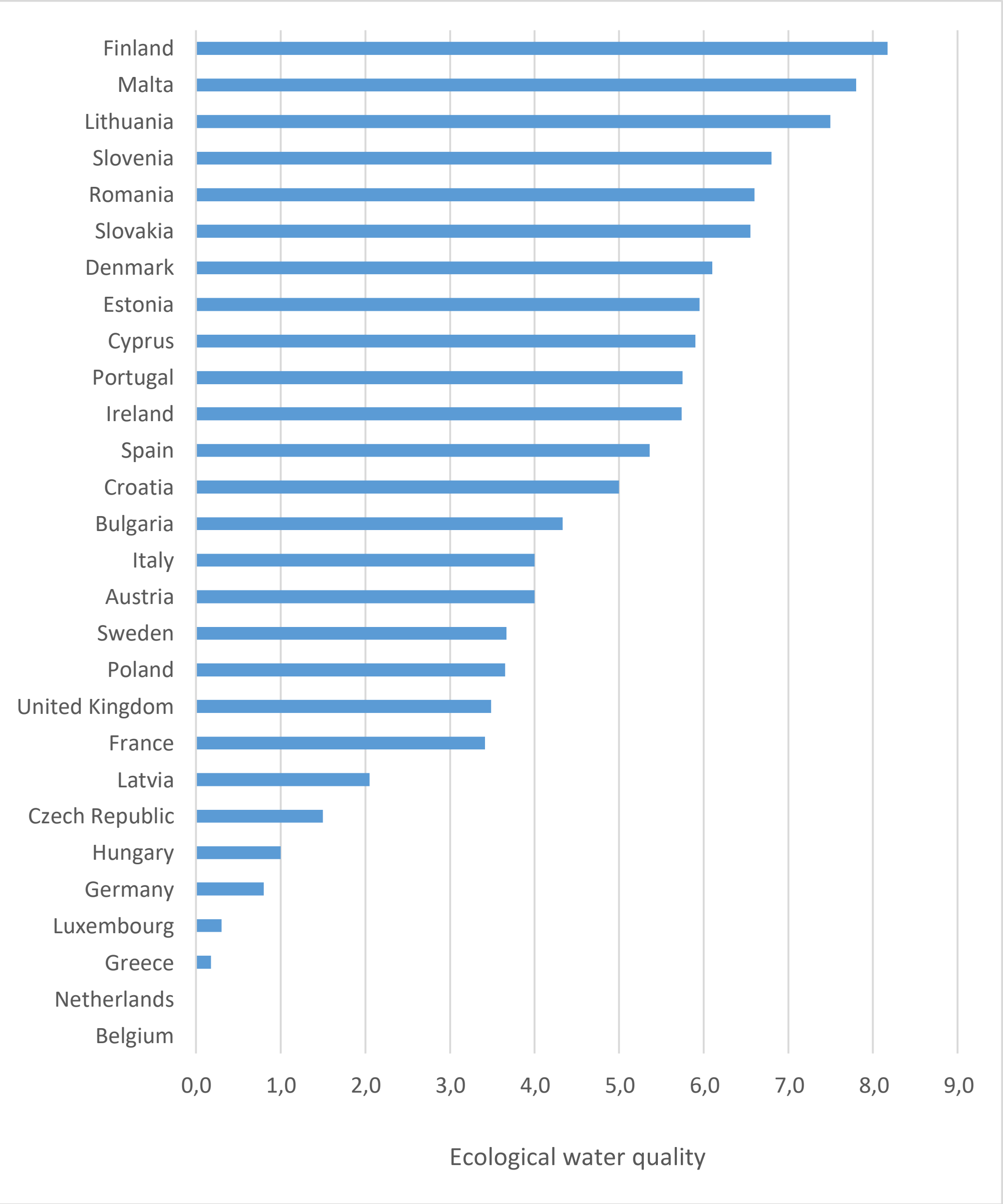

8. Connected to drinking water supply (%)

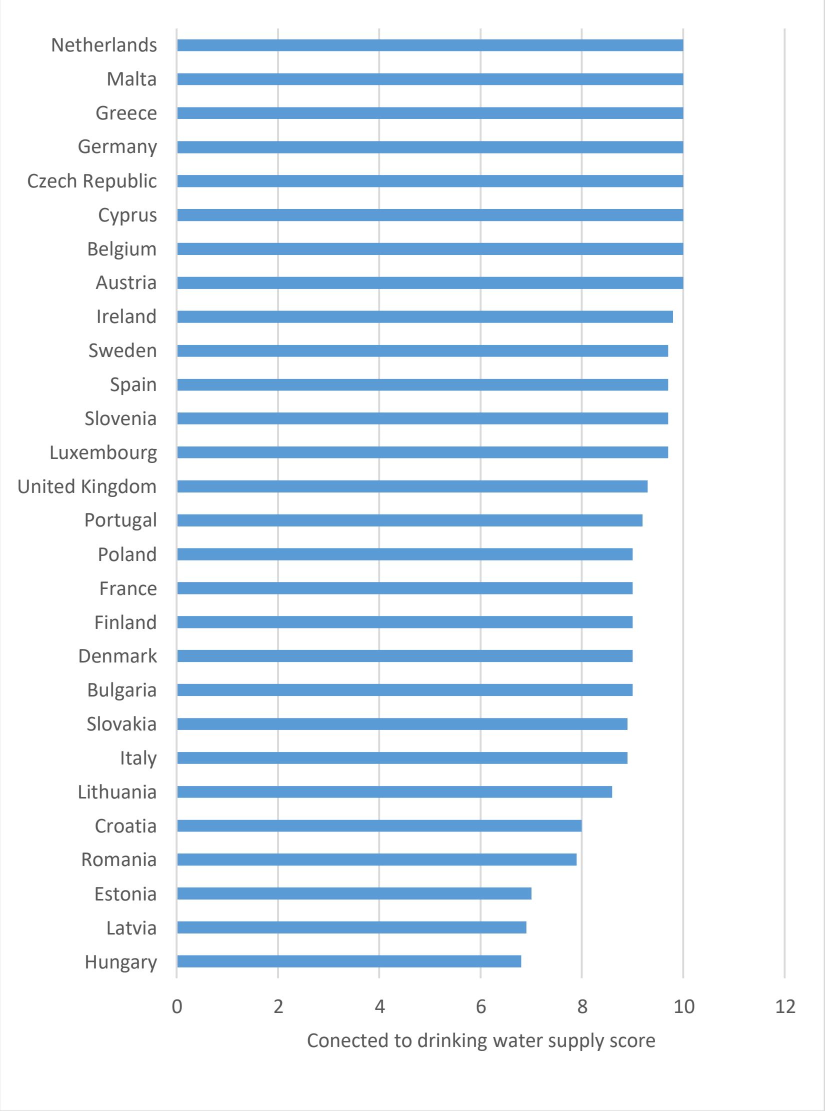

9. Connected to improved sanitation (%)

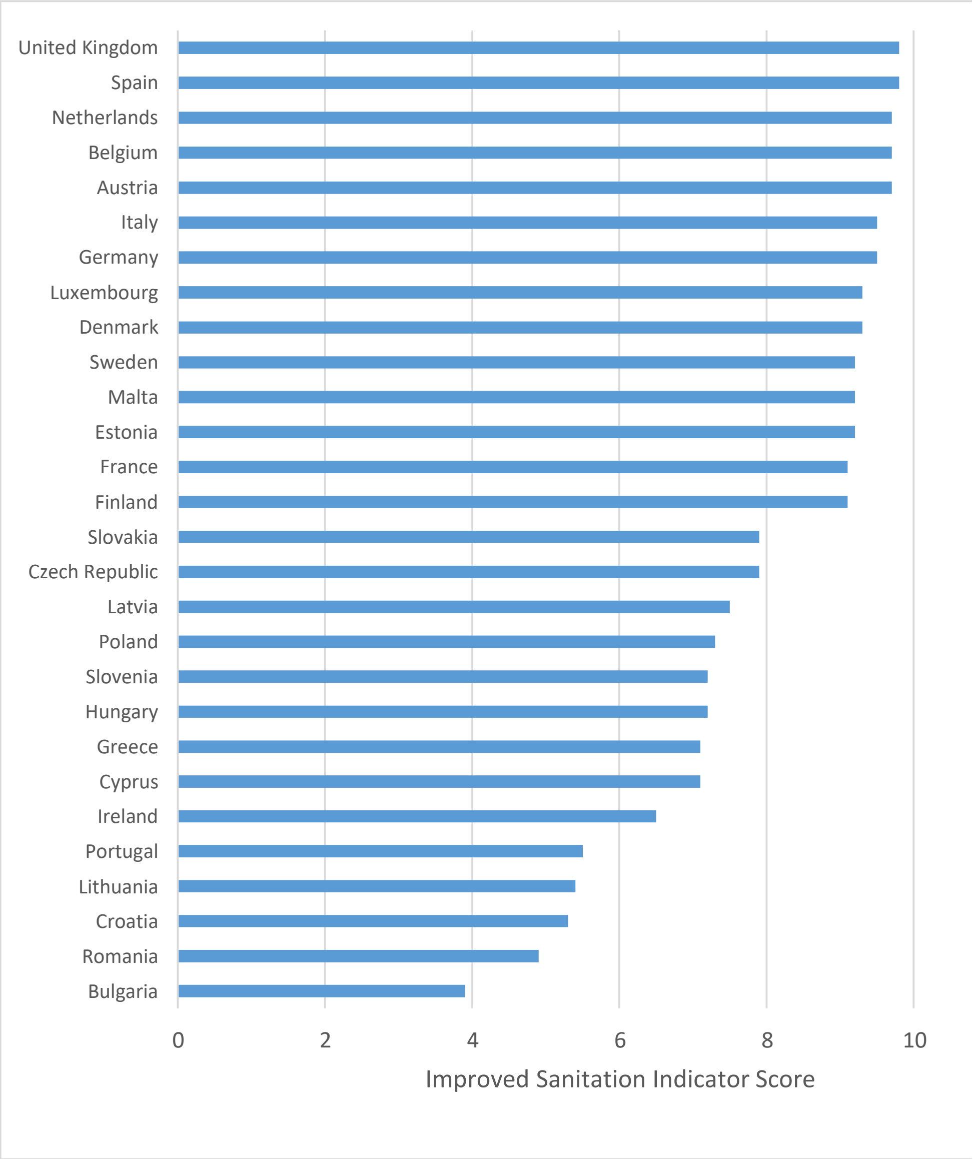

13. Secondary WWT

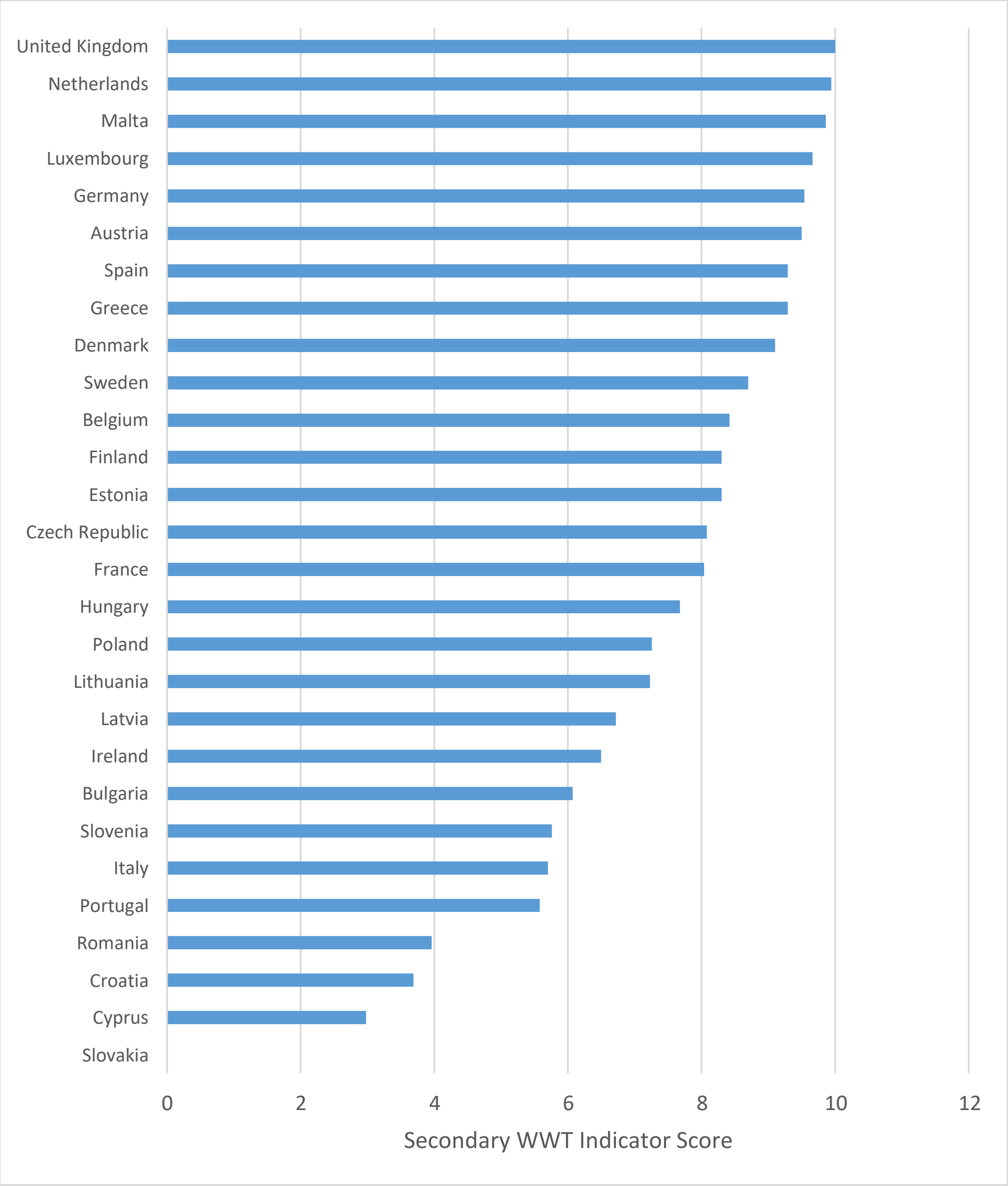

24. Indicator score implementation of IWRM

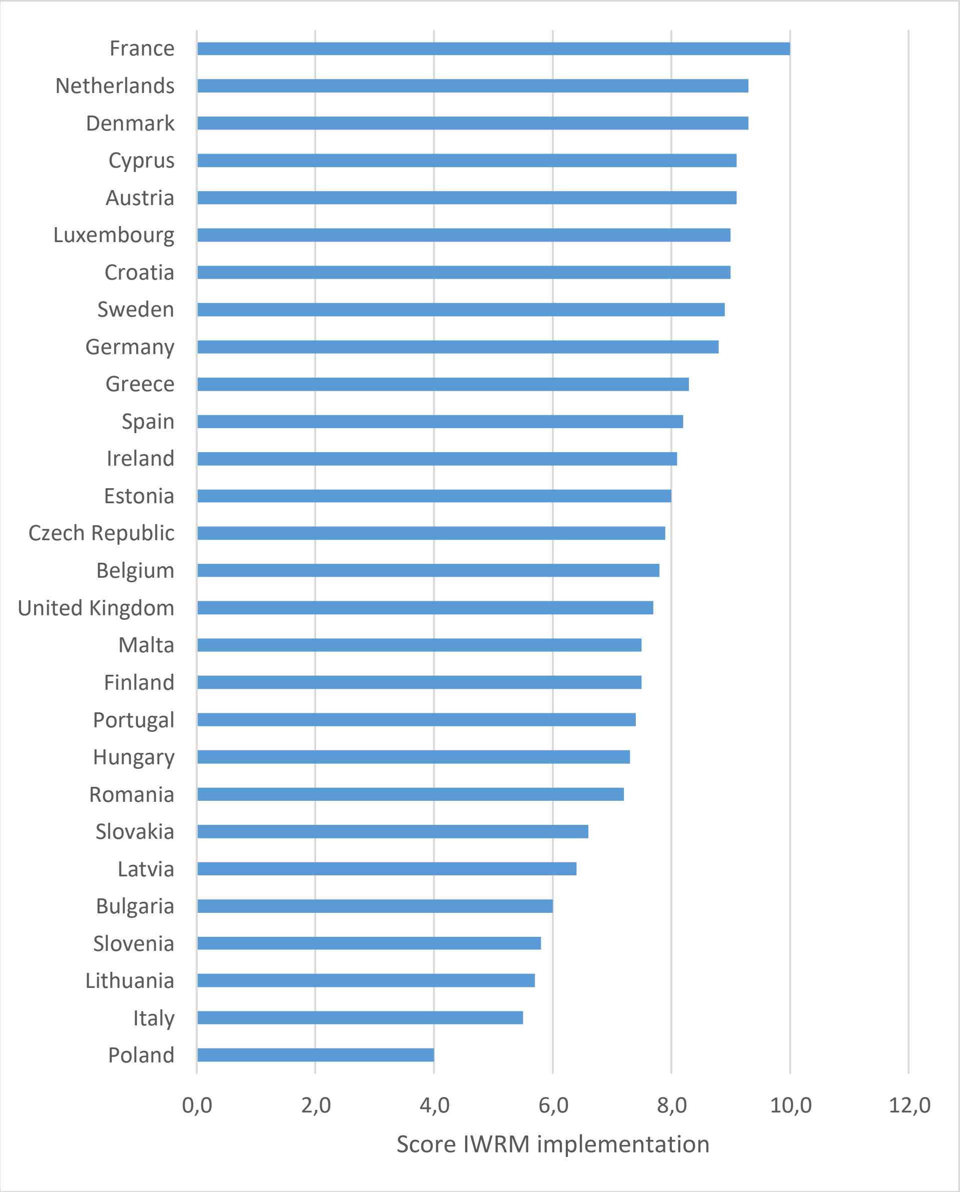

ANNEX 3. SPIDER DIAGRAMS OF THE NBF FOR EACH OF THE 28 EU MEMBER STATES

The following section gives the 24 indicator scores for each country in a spider diagram.

Austria

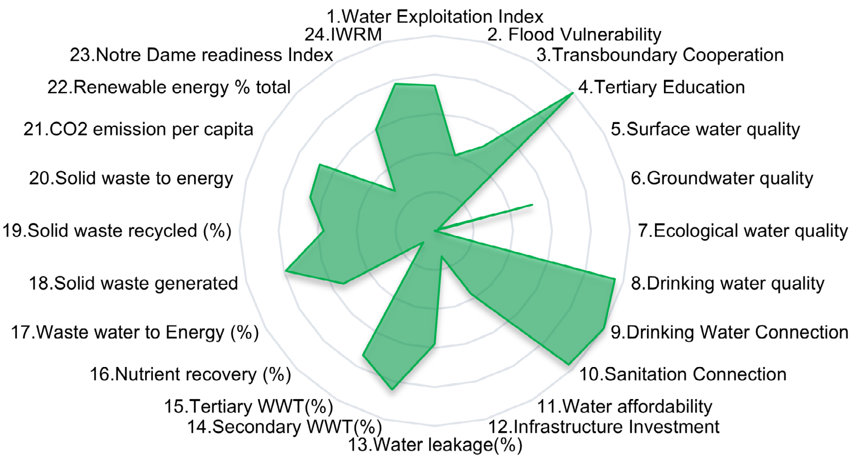

Belgium

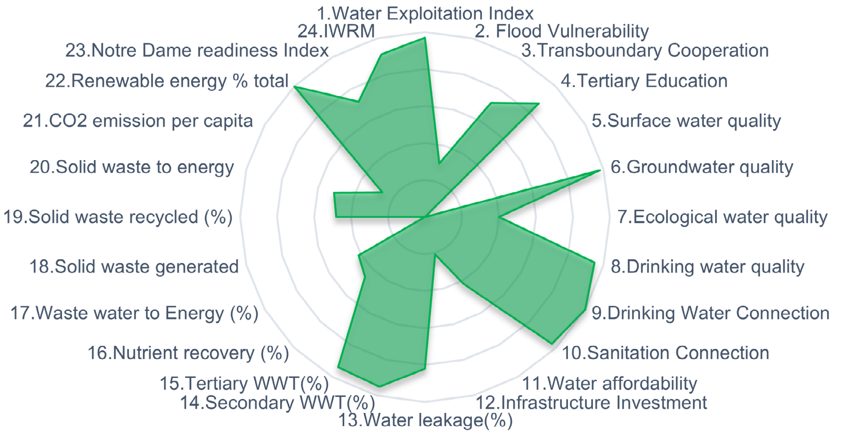

Bulgaria

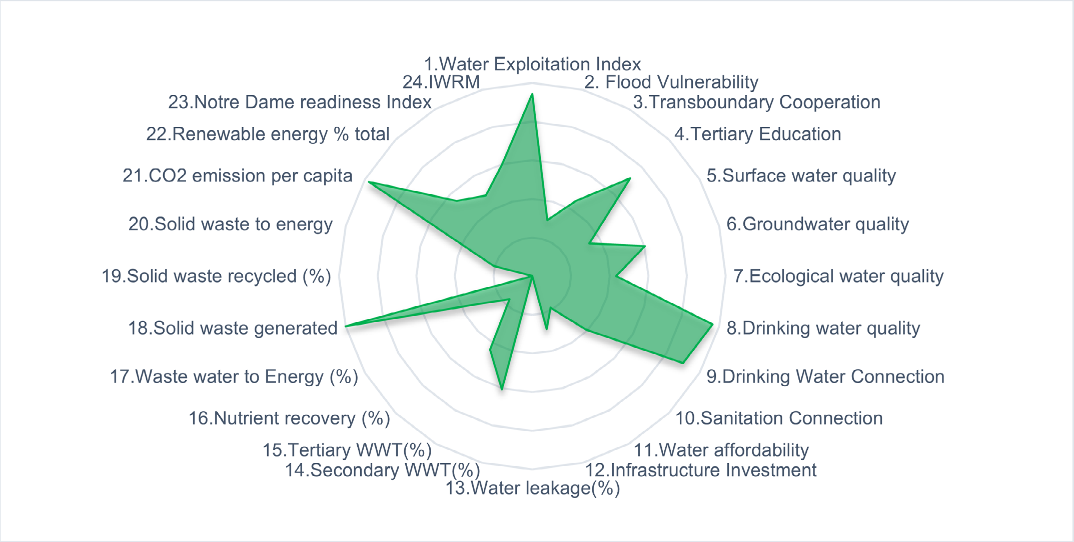

Croatia

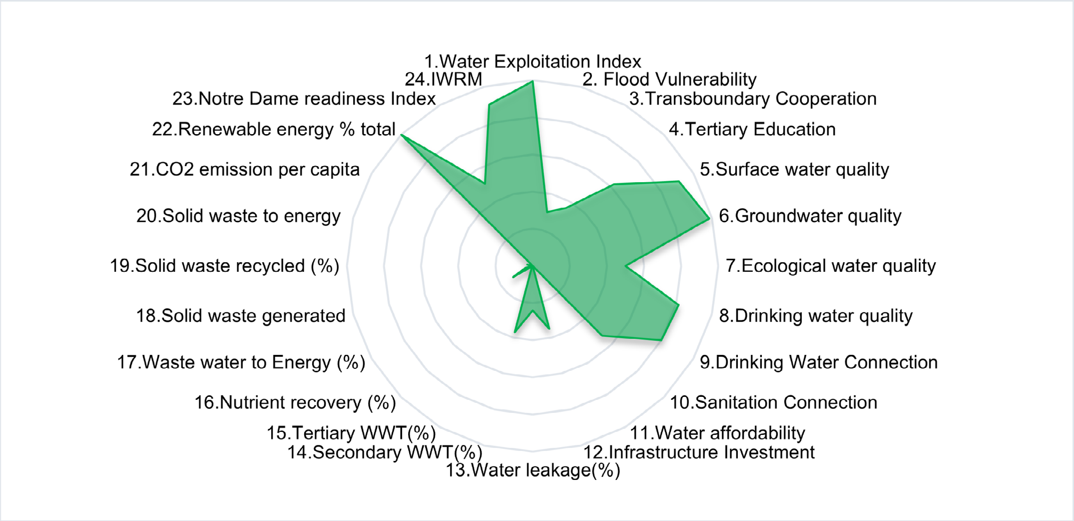

Cyprus

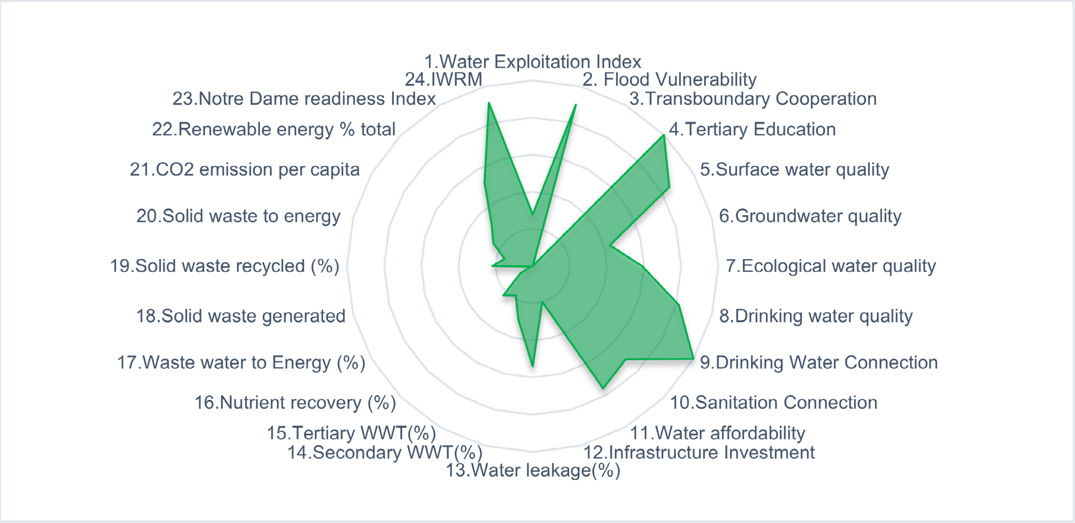

Czech Republic

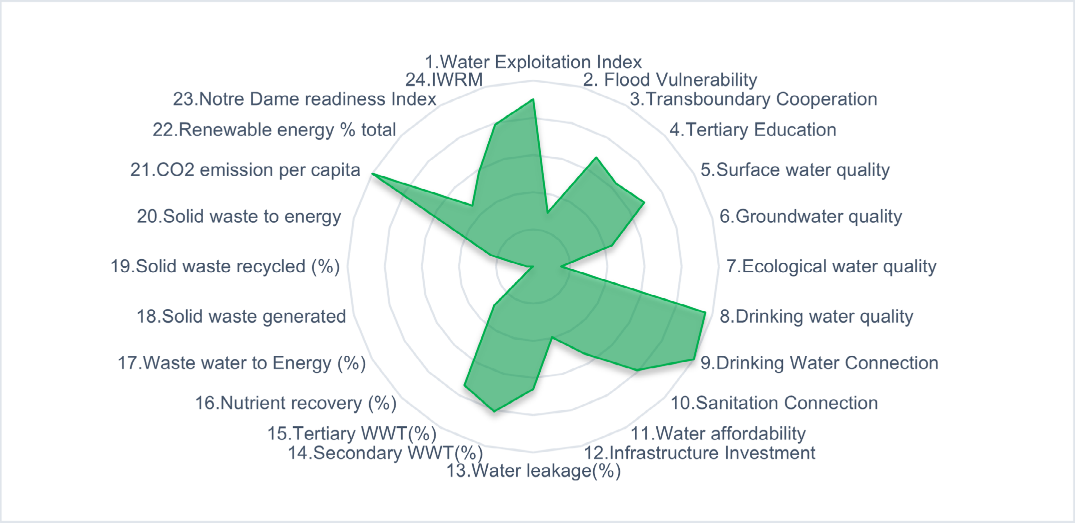

Denmark

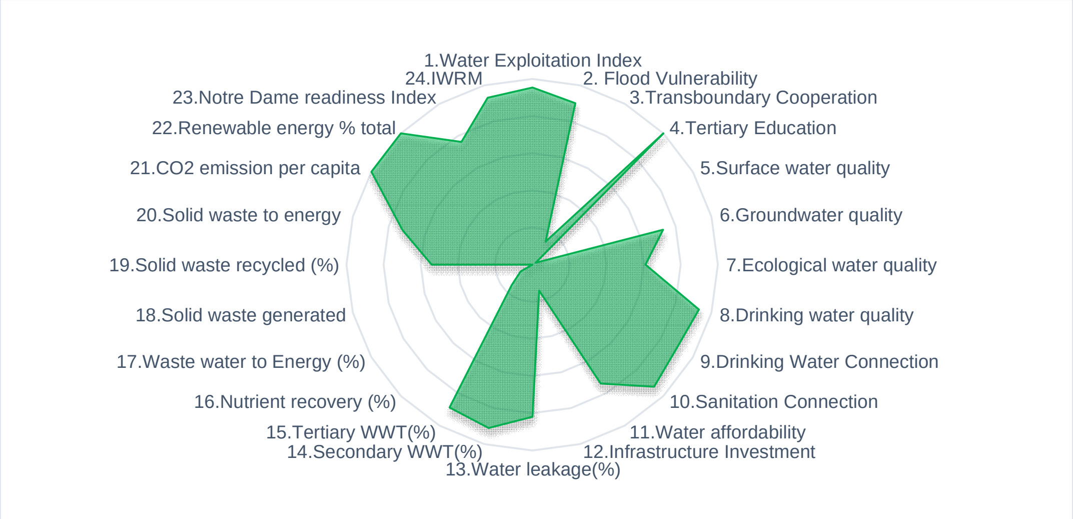

Estonia

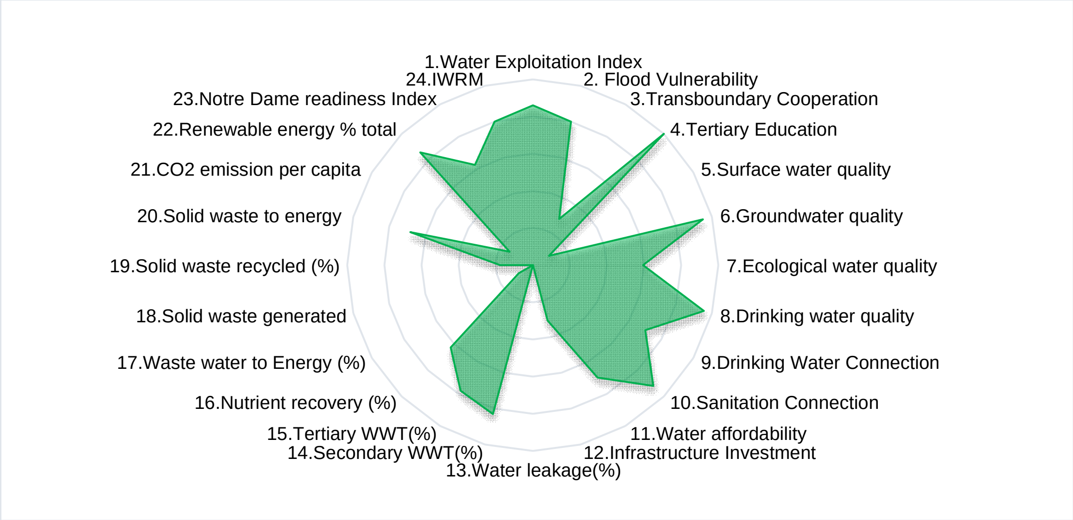

Finland

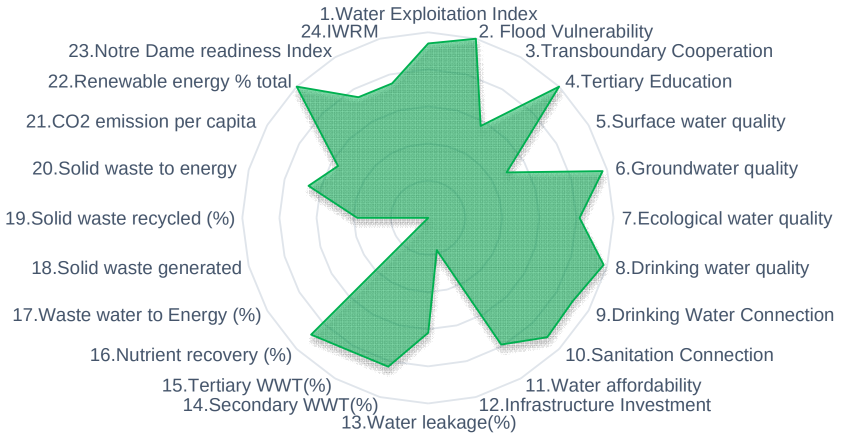

France

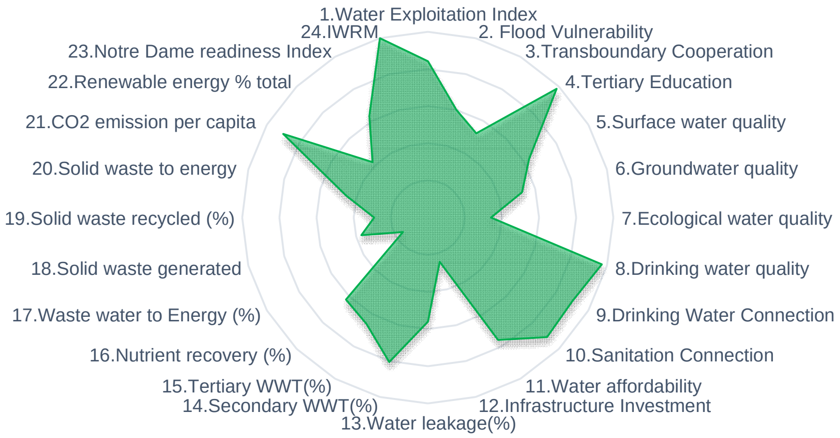

Germany

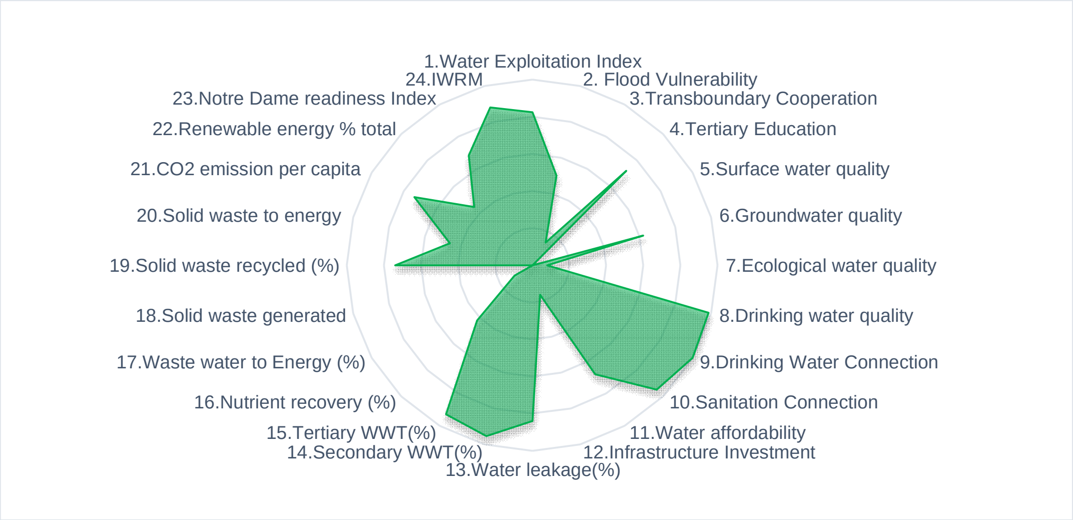

Greece

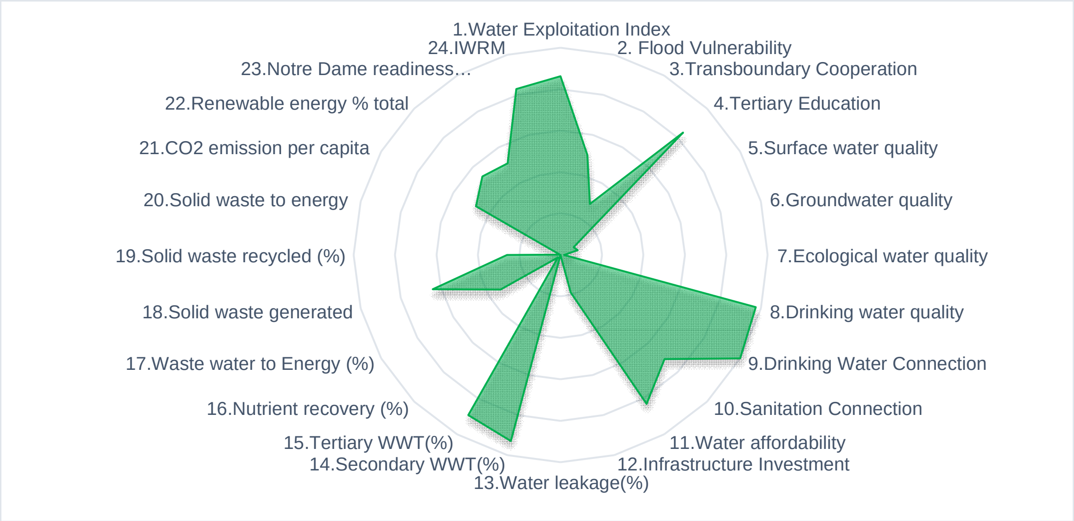

Hungary

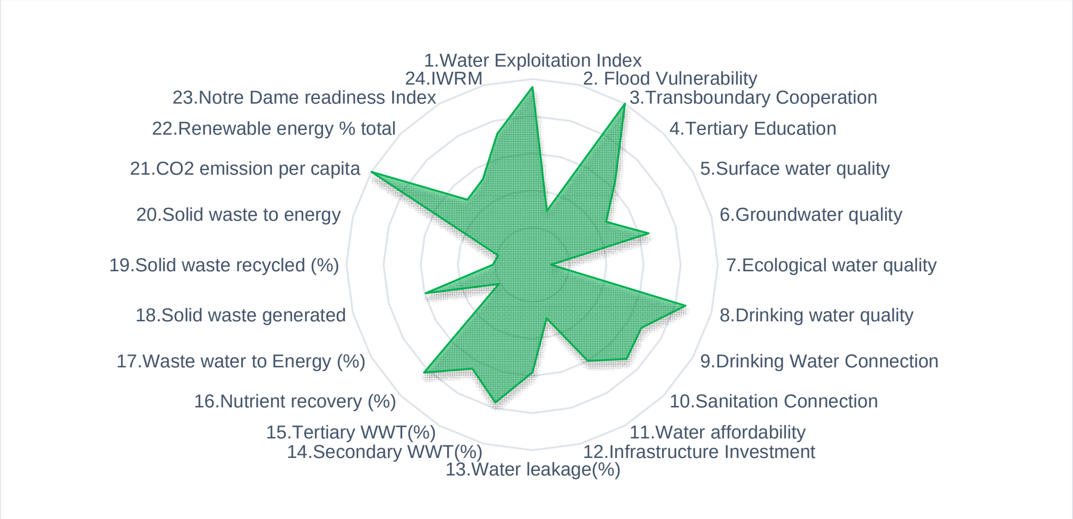

Ireland

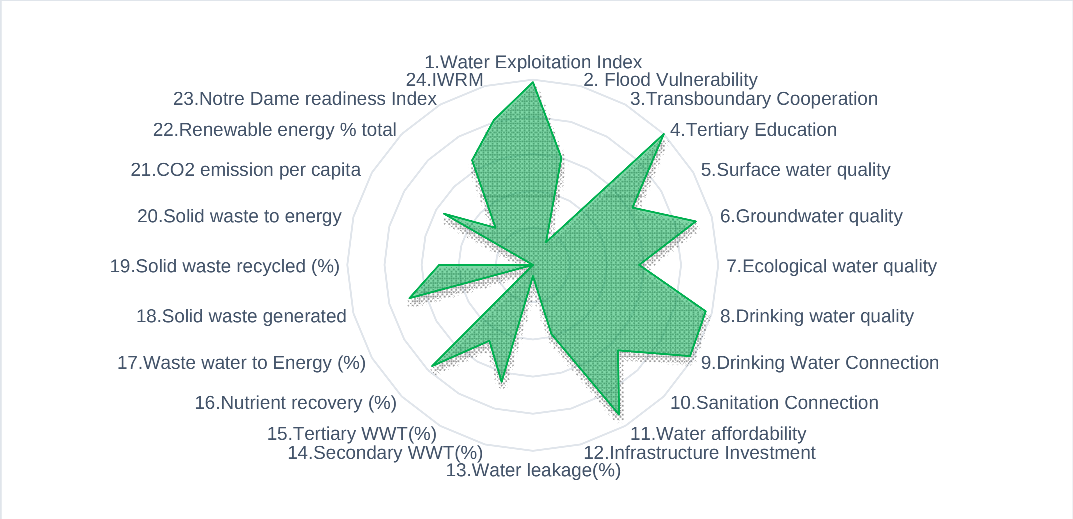

Italy

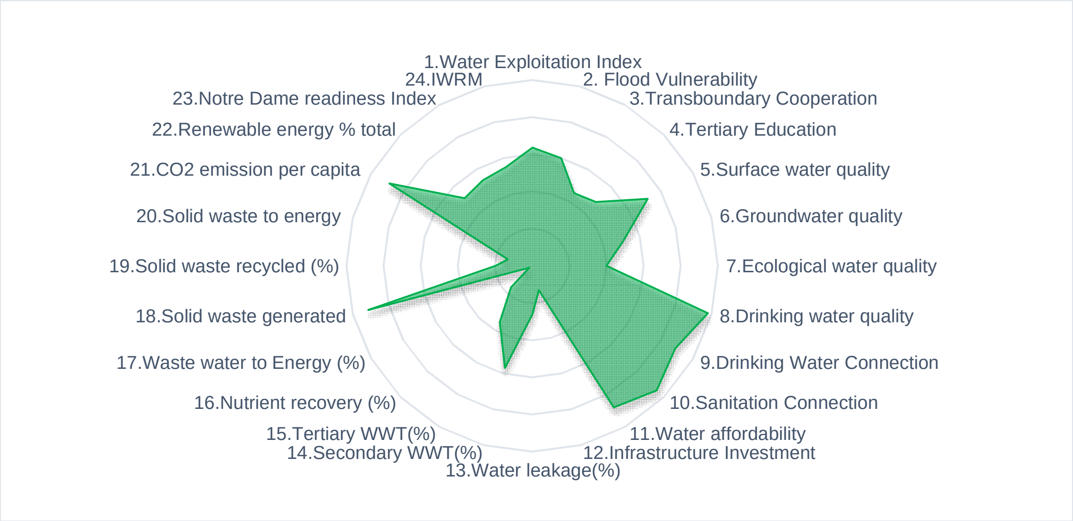

Latvia

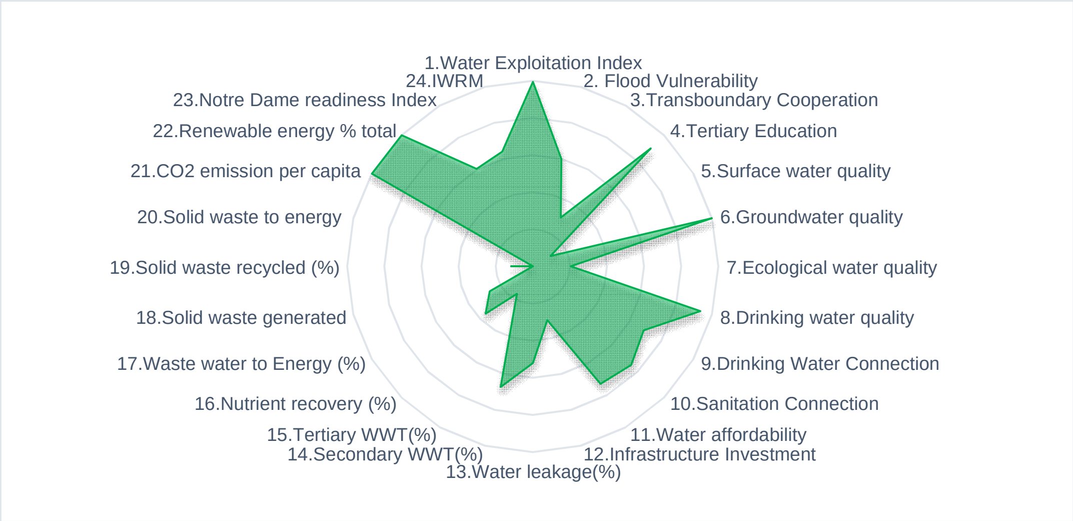

Lithuania

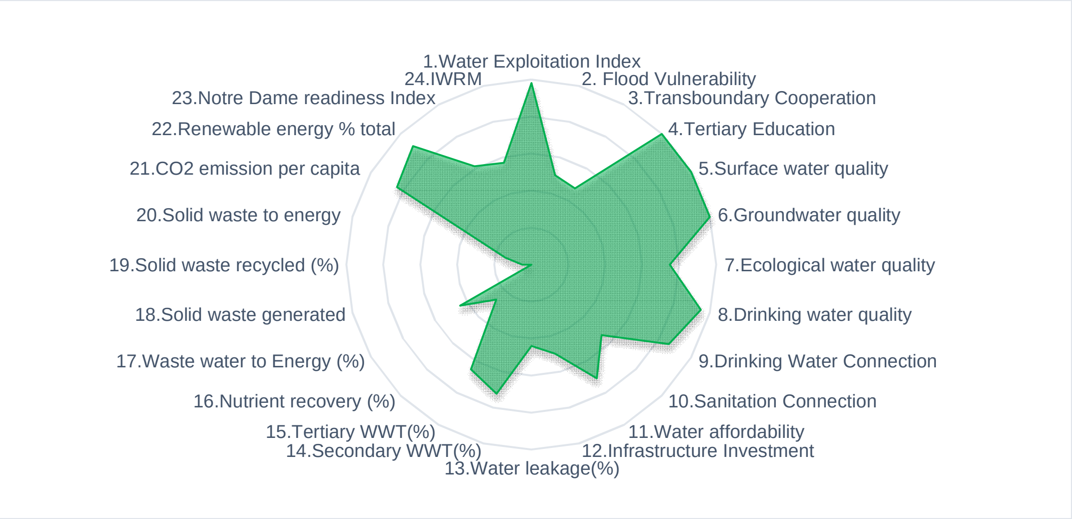

Luxembourg

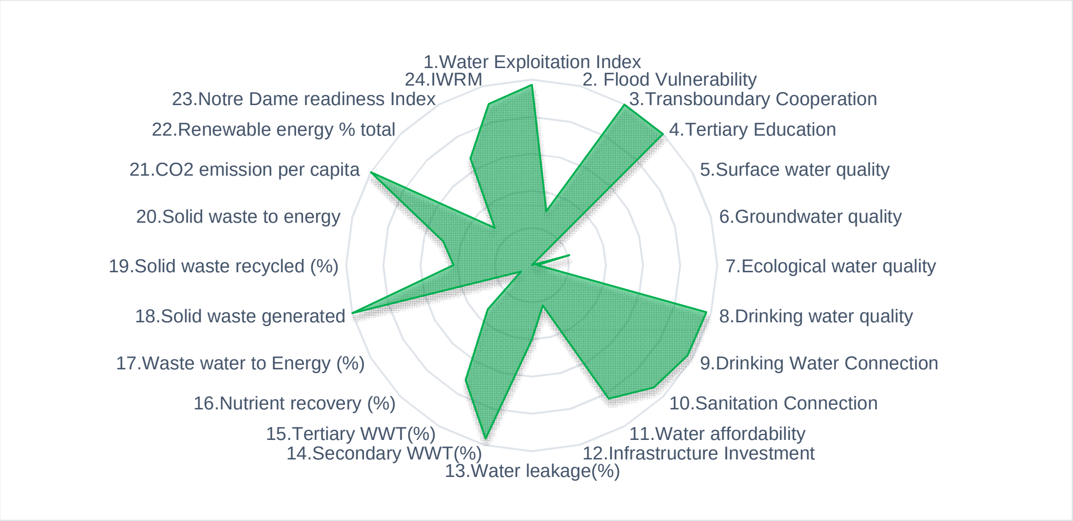

Malta

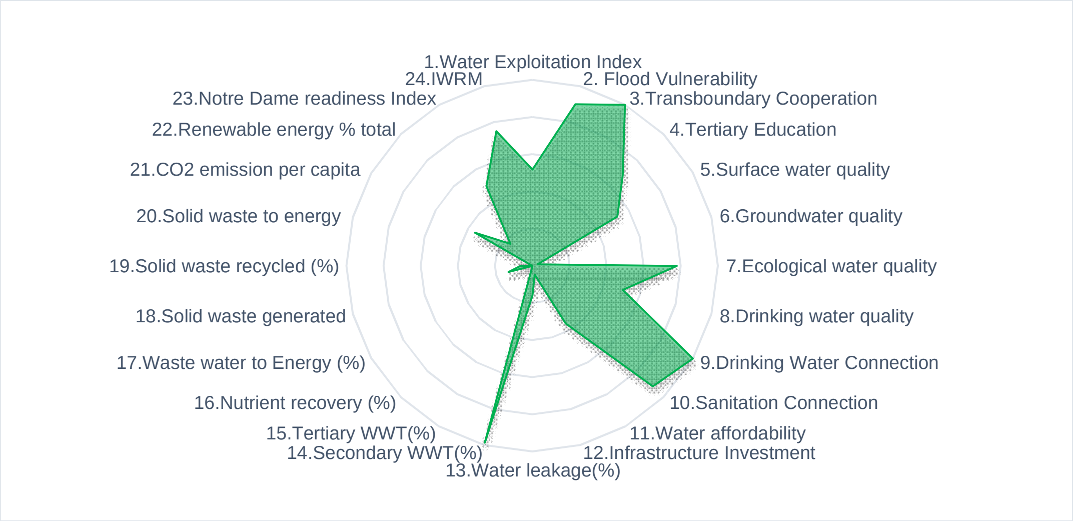

Netherlands

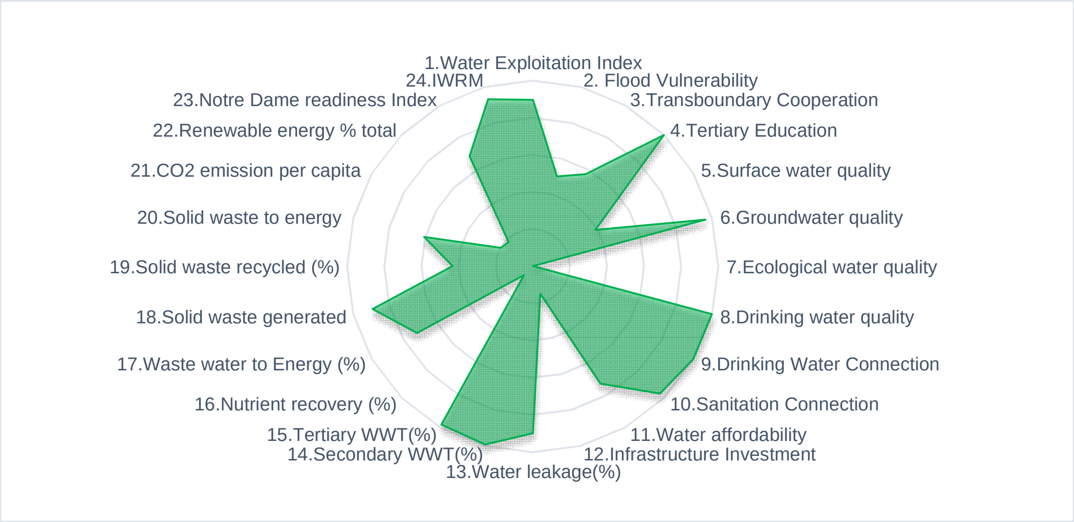

Poland

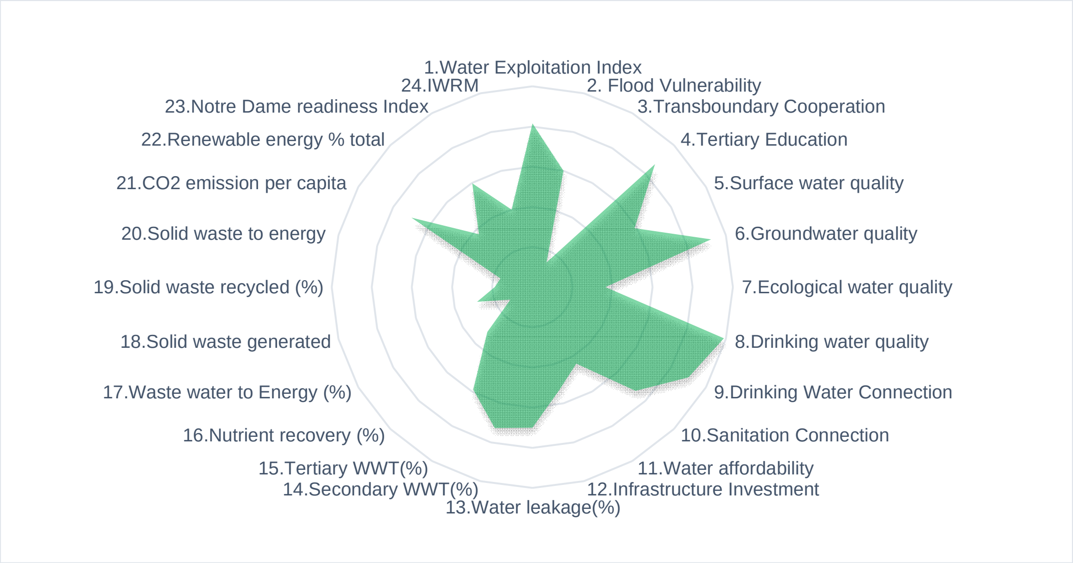

Portugal

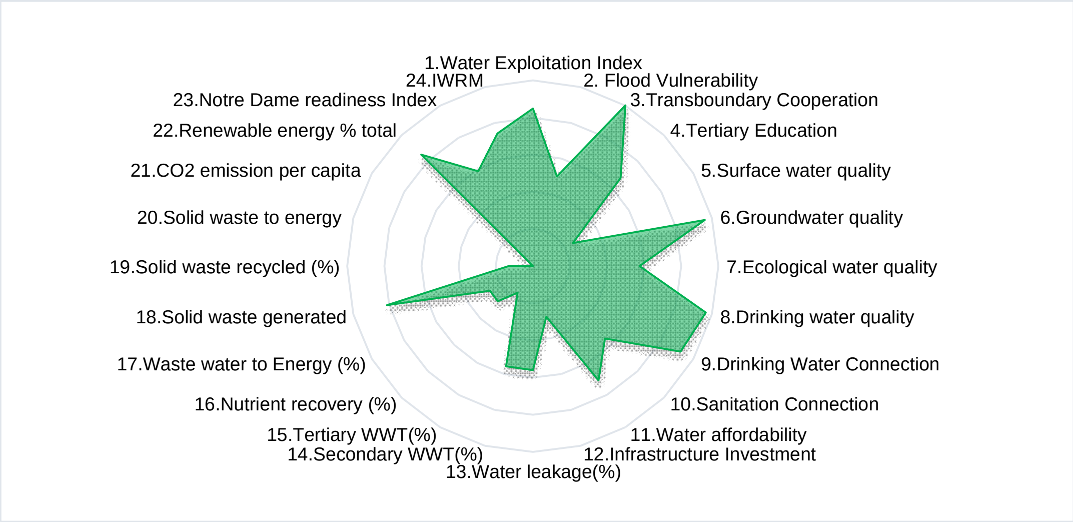

Romania

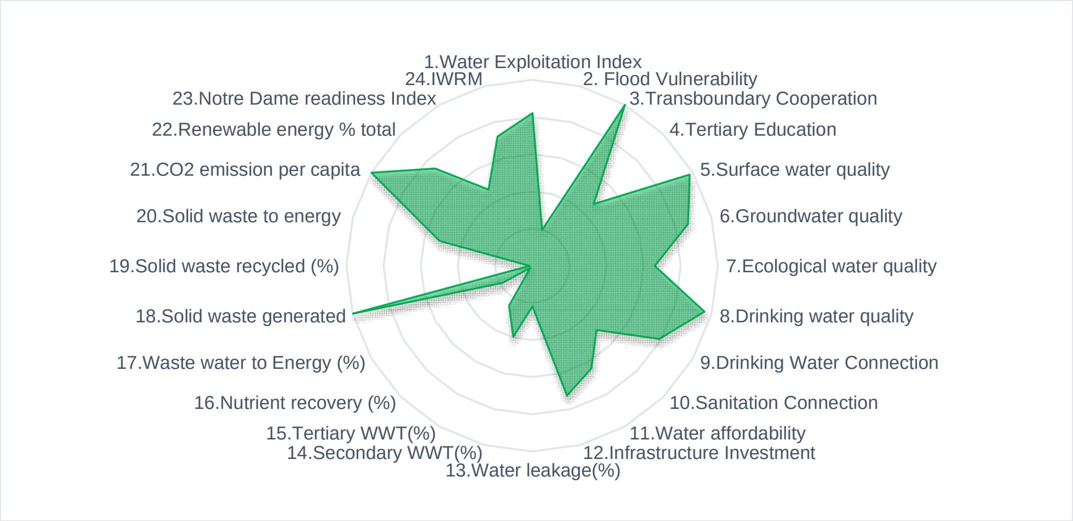

Slovakia

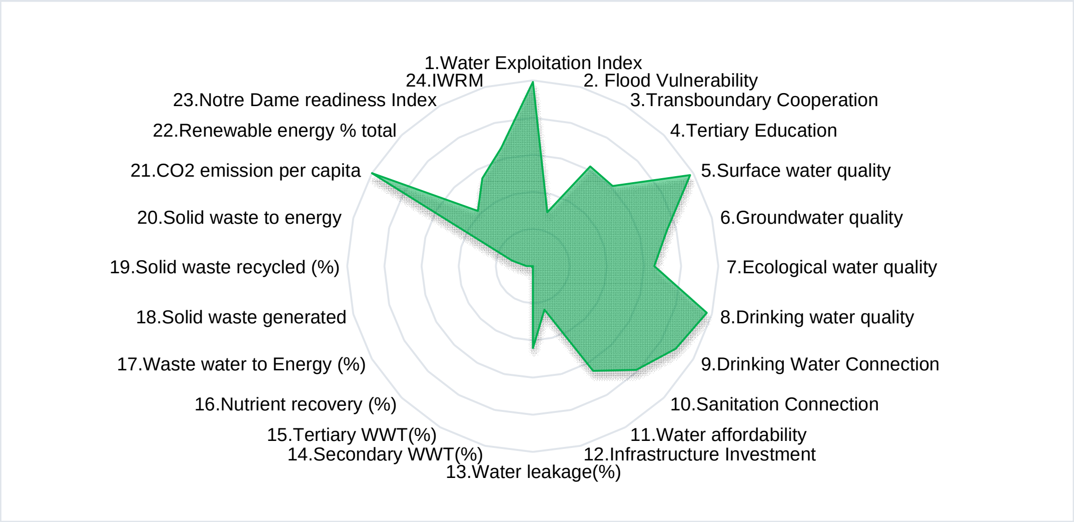

Slovenia

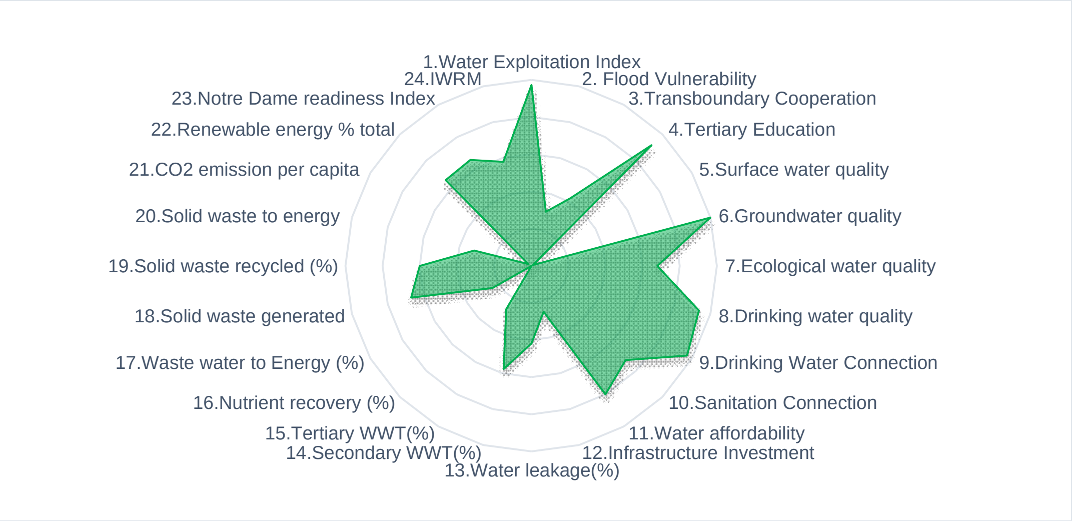

Spain

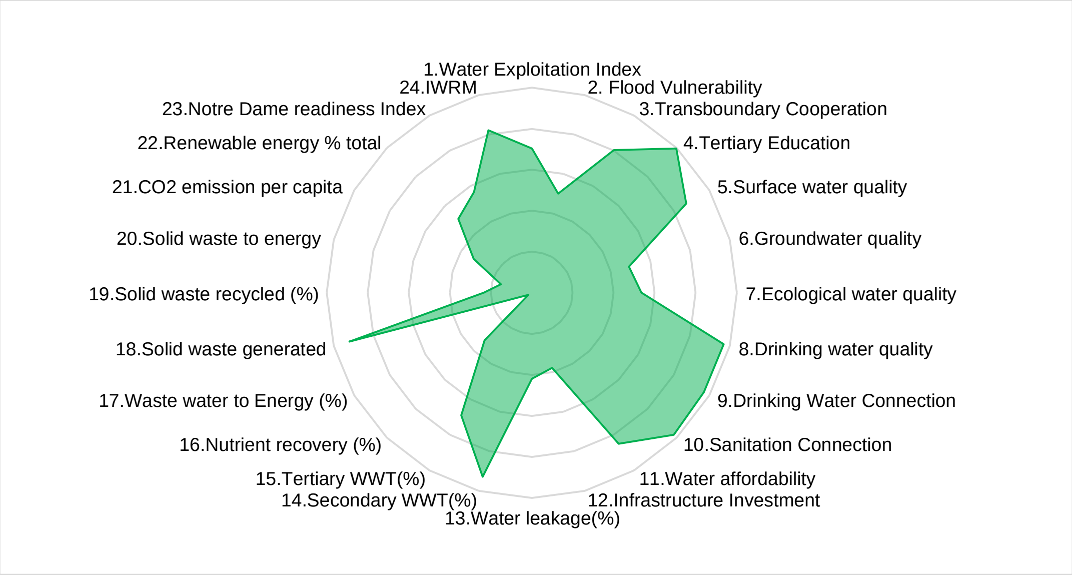

Sweden

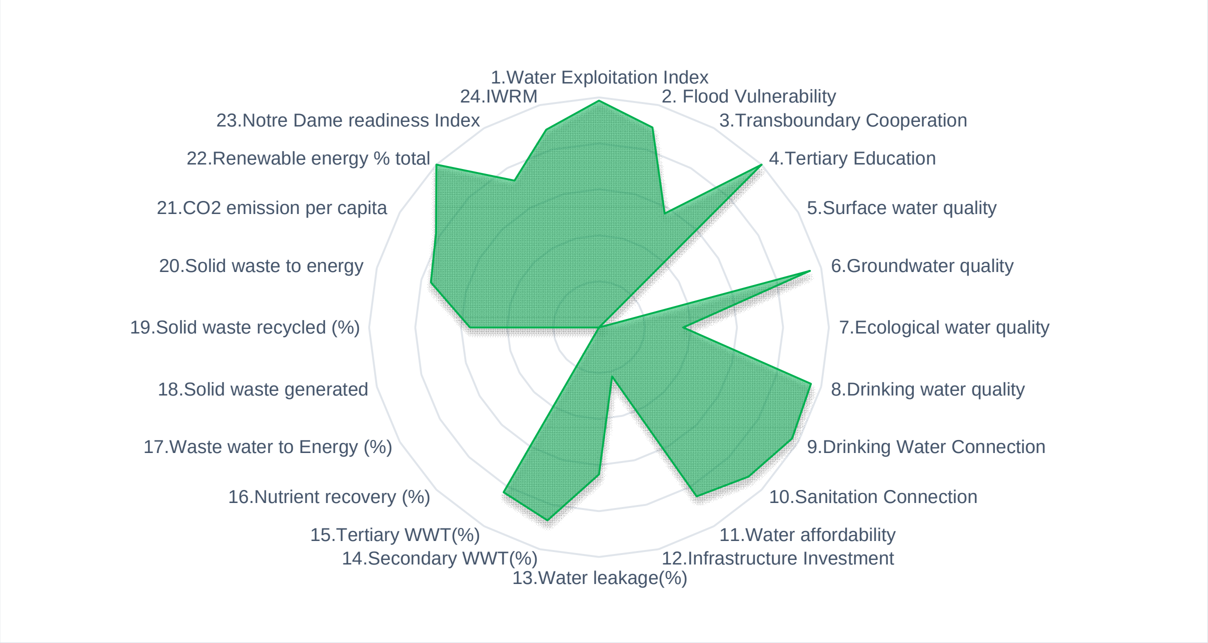

United Kingdom

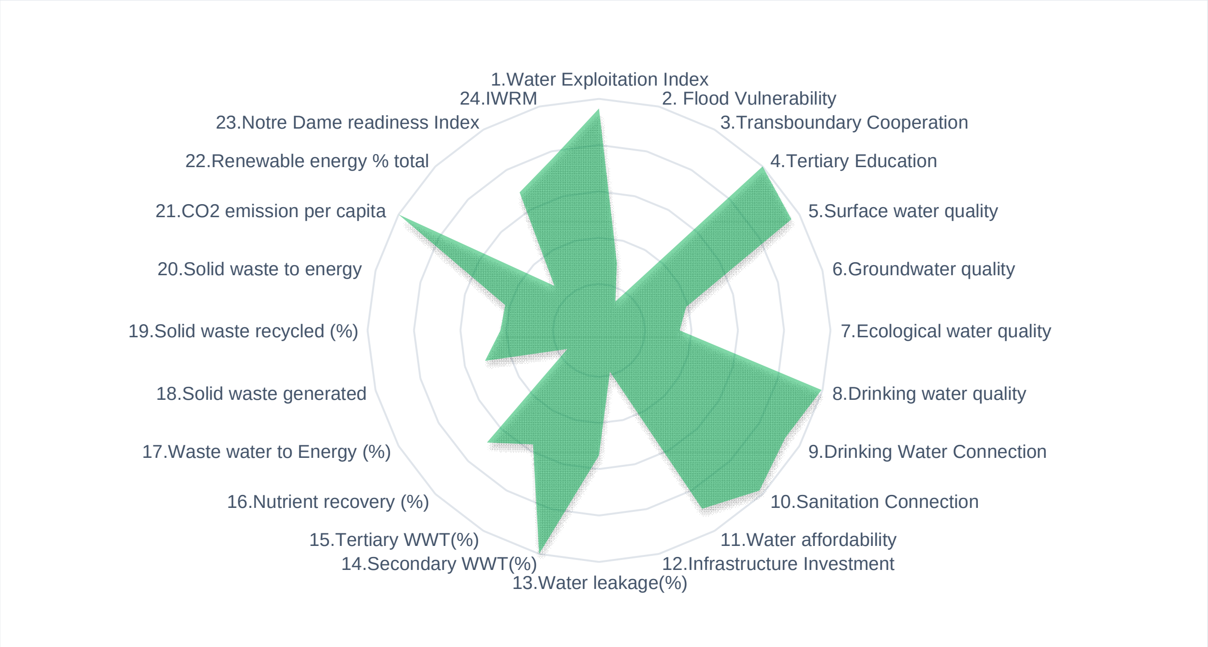

Supplement: Supplementary file 1 — Supplementary material [file 267_2019_1231_MOESM1_ESM.pdf]
